# Physics‐Informed Neural Network‐Enabled Forward Prediction and Inverse Design of Ring Origami

**DOI:** 10.1002/advs.76194

**Published:** 2026-06-22

**Authors:** Luyuan Ning, Lu Lu, Sophie Leanza, Ruike Renee Zhao

**Affiliations:** ^1^ Department of Mechanical Engineering Stanford University Stanford California USA

**Keywords:** forward prediction, inverse design, physics‐informed neural network, ring origami, shape morphing

## Abstract

Ring origami, consisting of closed‐loop rods, can realize diverse shape‐morphing behaviors, including 2D‐to‐1D, 2D‐to‐2D, and 2D‐to‐3D transformations, by harnessing snap‐buckling instability. To broaden its application potential in areas such as deployable aerospace structures, soft robotics, and reconfigurable metamaterials, a programmable design framework is highly desired. In this work, we develop a unified framework for the forward prediction and inverse design of ring origami by integrating Kirchhoff rod theory with a physics‐informed neural network. The framework can identify the stable states of various segmented rings (e.g., square and hexagonal rings) composed of rod segments with prescribed constant or varying natural curvature (i.e., curvature in the stress‐free state). By introducing an additional shape‐matching loss, the framework can also determine the natural curvature profile of segmented rings required to achieve stable configurations that can be confined within a target spatial domain or conform to a target curved surface. Its generality and robustness are further demonstrated by extending it to 3D rod systems. This work establishes a powerful strategy for the programmable design of elastic rod systems exemplified by ring origami and opens new opportunities for functional applications that demand shape‐morphing structures with simple geometries, high packing capability, and prescribed stable configurations.

## Graphical abstract

This work presents a KRT‐PINN framework that integrates Kirchhoff rod theory with physics‐informed neural networks for the forward prediction and inverse design of ring origami consisting of closed‐loop rods. The framework predicts stable states of segmented rings with prescribed natural‐curvature profiles and determines the natural‐curvature profiles required to achieve target stable configurations, enabling the programmable design of shape‐morphing elastic rod systems.

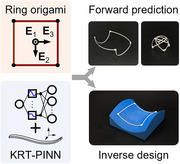

## 1 Introduction

**FIGURE 1:**
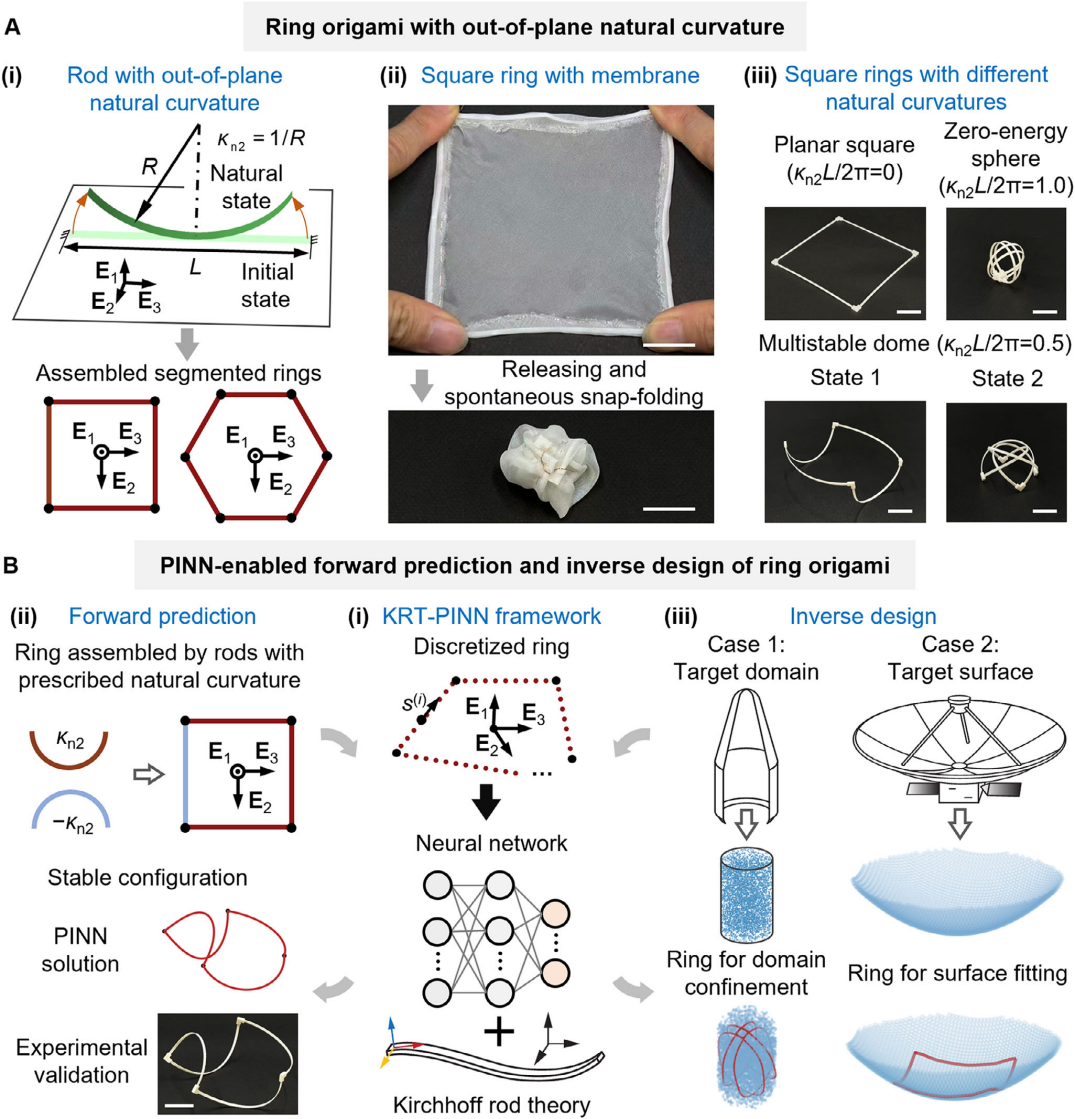
PINN‐enabled design framework for ring origami. (A) Ring origami with out‐of‐plane natural curvature: (i) Schematic of a rod segment with out‐of‐plane natural curvature *κ*_n2_ = 1/*R*, which can be elastically straightened and then assembled into segmented rings. (ii) Spontaneous snap‐folding of a square ring with a membrane upon releasing the external constraints. Scale bars: 20 mm. (iii) Stable configurations of square rings with different out‐of‐plane natural curvatures. Scale bars: 20 mm. (B) PINN‐enabled forward prediction and inverse design of ring origami: (i) Schematic of the proposed KRT‐PINN framework. (ii) Forward prediction of ring origami consisting of rod segments with prescribed natural curvature. (iii) Inverse design of the stable configuration of ring origami confined within a target spatial domain or conforming to a target curved surface.

Ring origami [1, 2], consisting of closed‐loop rods, has recently emerged as a powerful platform for designing shape‐morphing structures [3, 4]. It has been shown that, under external stimuli, ring origami, such as circular [5, 6] and polygonal rings [7, 8], can fold into compact configurations with extreme packing ratios by harnessing snap‐buckling instability, enabling 2D‐to‐1D or 2D‐to‐2D transformation [5, 6, 7, 8, 9, 10]. Recent studies have further demonstrated that by introducing out‐of‐plane natural curvature into the rod segments (i.e., the stress‐free state lies in a plane perpendicular to the planar ring, as shown in Figure 1A‐i), a planar segmented ring (e.g., a square or hexagonal ring) can spontaneously transform into a 3D stable state through the natural curvature‐induced snapping, thereby enabling 2D‐to‐3D transformation [11, 12]. This behavior is exemplified by a square ring with a membrane in Figure 1A‐ii, where the ring spontaneously snaps‐folds into the compact state once the external constraints are removed. By programming the natural curvature and rod segment number, 2D segmented rings can morph into a wide range of 3D configurations [11]. For example, a planar square ring with rationally selected out‐of‐plane natural curvature can transform into a zero‐energy sphere or a multistable dome (Figure 1A‐iii). These rich shape‐morphing behaviors enabled by simple ring geometries make ring origami a promising candidate for various functional applications, including deployable aerospace structures [13, 14, 15], soft robotics [16, 17, 18], and reconfigurable metamaterials [19, 20, 21].

The elastic stability and shape‐morphing behavior of ring origami significantly depend on the natural curvature, cross‐sectional aspect ratio, and number of rod segments [5, 8, 22, 23]. These design parameters offer a vast design space for ring origami to realize diverse 3D stable configurations [11]. The high degree of programmability also makes ring origami a versatile platform for the inverse design of morphing structures to achieve target stable configurations, which is particularly attractive in engineering applications requiring prescribed shapes based on simple geometries. For example, space antennas often employ parabolic geometries to improve signal focusing and reflection [24]. However, the complex nonlinear behavior and geometric discontinuities of ring origami, such as curvature discontinuities at the joints of segmented rings, often result in a system of ordinary differential equations with a large number of unknowns [7, 25], making forward simulation of its natural curvature‐dependent mechanical behavior computationally expensive. As a result, inverse design of ring origami for target stable configurations remains challenging.

Physics‐informed neural networks (PINNs) [26, 27, 28] provide a promising approach to address these challenges by directly integrating governing equations into neural network training, thereby enabling the equilibrium configuration and design variables to be optimized within a unified differentiable framework without generating a supervised training dataset and with reduced reliance on repeated forward simulations in inverse design workflows. As a general framework for solving nonlinear partial differential equations, PINNs have been widely applied and proven to be effective in characterizing the nonlinear behavior of diverse solid mechanics systems [29, 30, 31, 32, 33]. Compared with purely data‐driven neural networks, PINNs provide better physical interpretability because the embedded governing equations in the loss function, and the neural network serves as a continuous representation of the solution field [34]. This advantage is particularly important for ring origami, in which large bending and twisting deformations, combined with the natural curvature‐induced internal stresses, can give rise to multiple equilibrium states and snapping transitions [11]. Consequently, the relationship between natural curvature and stable configurations becomes non‐unique and difficult to learn using purely data‐driven approaches. A physics‐guided framework is therefore especially well‐suited to this problem, as it enables direct solution of the nonlinear equilibrium equations of ring origami under mechanical constraints.

To this end, we integrate Kirchhoff rod theory (KRT) [35], which governs the mechanical behavior of slender elastic rods, within a PINN to develop a unified design framework, termed the KRT‐PINN framework (Figure 1B‐i), for the forward prediction and inverse design of ring origami composed of rod segments with natural curvature. In the forward prediction (Figure 1B‐ii), the stable state of a segmented ring with a prescribed natural curvature profile is efficiently determined by minimizing the total potential energy. By employing randomized initializations of the ring configuration, different stable states can be identified. In the inverse design, the natural curvature profile of segmented rings is determined to achieve a target stable configuration, such as being confined within a target spatial domain (case 1 in Figure 1B‐iii) or conforming to a target curved surface (case 2 in Figure 1B‐iii), enabled by introducing a shape‐matching loss augmented with a penalty function method. Both the forward prediction and inverse design are experimentally validated, showing good agreement. The generality and robustness of the proposed framework are further demonstrated by extending it to 3D rod systems, where it successfully predicts the various stable configurations of tetrahedra and cubes assembled from rod segments with natural curvature. These results establish the proposed KRT‐PINN framework as a powerful tool for the programmable design of elastic rod systems represented by ring origami, with promising potential for functional applications where shape‐morphing structures with simple geometries, high packing capability, and prescribed stable configurations are required.

## 2 Results

### 2.1 The KRT‐PINN Framework

**FIGURE 2:**
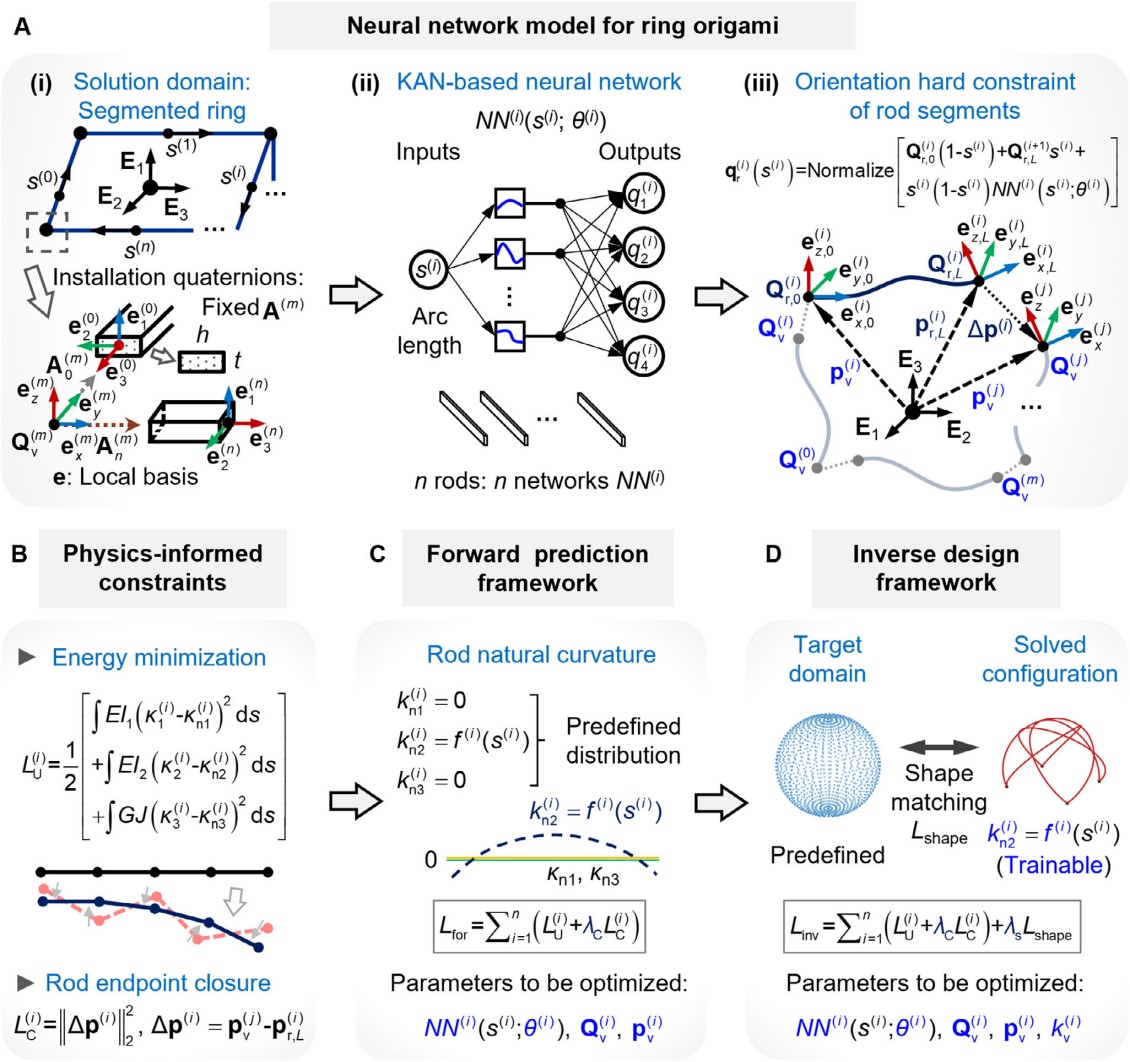
The KRT‐PINN framework for forward prediction and inverse design of ring origami. (A) Neural network model for ring origami: (i) Solution domain of a segmented ring, parameterized by the arc length *s* ^(*i*)^ and fixed installation quaternions $A_{k}^{\left(m\right)}$. (ii) The KAN‐based neural network, which maps the normalized arc length to local temporary quaternions **q** ^(*i*)^. (iii) Orientation hard constraint and closure condition for individual rod segments, which enforces rotational continuity in the segmented ring. (B) Physics‐informed constraints of the framework, including the rod potential energy $L_{U}^{\left(i\right)}$ based on KRT and the rod endpoint closure condition $L_{C}^{\left(i\right)}$. (C) Forward prediction framework: The loss function is defined by $L_{U}^{\left(i\right)}$ and $L_{C}^{\left(i\right)}$. For a ring with a prescribed natural curvature profile (*κ*_n1_, *κ*_n2_, *κ*_n3_), its stable state can be identified by optimizing the network parameters, vertex position vector, and vertex orientations. (D) Inverse design framework: The loss function is defined by $L_{U}^{\left(i\right)}$ and $L_{C}^{\left(i\right)}$, together with an additional shape‐matching loss *L*_shape_. For a prescribed target spatial domain or curved surface, the natural curvature profile required for the ring to be confined within the target domain or conform to the target surface can be determined by optimizing the network parameters, vertex position vector, vertex orientations, and natural curvature.

We begin by introducing the KRT‐PINN framework, which provides a unified approach for the forward prediction and inverse design of ring origami consisting of rod segments with natural curvature. First, a neural network model for segmented rings is constructed, in which Kolmogorov‐Arnold Networks (KANs) [36] are employed to learn the mapping between ring design parameters and the corresponding ring configurations. The neural network model is then integrated with physics‐informed constraints to define the loss functions, resulting in a physics‐informed optimization framework for both forward prediction and inverse design. The overall workflow of the KRT‐PINN framework is illustrated in Figure 2.

As shown in Figure 2A‐i, the segmented ring is assembled by rod segments with the same length *L* and rectangular cross‐section of width *t* and height *h*. The rod segments are assembled by rigid joints. To represent the rigid connections between adjacent rods, the joints are modeled as fixed rigid transformations between the local frames $\left(e_{1}^{\left(i\right)},e_{2}^{\left(i\right)},e_{3}^{\left(i\right)}\right)$ of the rods and the vertices, where *i* = 1, 2, ⋯ denotes the *i*‐th rod segment. Each vertex carries independent degrees of freedom, including a position vector $p_{v}^{\left(m\right)}$ and a quaternion orientation $Q_{v}^{\left(m\right)}$, where *m* = 1, 2, ⋯ represents the *m*‐th vertex. Here, the position vector is defined in the global frame (**E**_1_, **E**_2_, **E**_3_), whereas quaternions are introduced to represent the rod orientation in the local frame with respect to the global frame [37, 38], which can avoid the singularities associated with Euler angle parameterizations. The orientation of each rod endpoint has a fixed installation angle between the rod frame and the vertex frame. Therefore, fixed installation quaternions **A** ^(*m*)^ are defined on each vertex to ensure correct assembling.

The KAN [36] is applied as the basic trial function in the KRT‐PINN framework to represent the configuration of each rod segment (Figure 2A‐ii). For a segmented ring consisting of *n* rod segments, we define *n* sub‐networks, *NN* ^(^ *^i^* ^)^ (*i* = 1, …, *n*), where each network maps the normalized arc length *s* ^(^ *^i^* ^)^ ∈ [0, 1] to the temporary quaternion **q** ^(*i*)^ = *NN* ^(*i*)^(*s* ^(*i*)^; *θ* ^(*i*)^). Here, *θ* ^(^ *^i^* ^)^ denotes the learnable parameters of the *i*‐th KAN.

Unlike conventional multi‐layer perceptrons (MLPs), which apply fixed neuron‐wise activation functions, KANs employ learnable univariate functions on edges, typically parameterized by B‐splines, allowing more accurate representation of sharp gradients and high‐frequency variations in nonlinear curvature distributions with fewer parameters [36, 39]. A systematic comparison between the MLP‐based and KAN‐based PINNs in terms of convergence behavior, prediction accuracy, training stability, and parameter efficiency for the current ring origami problem is provided in Section 4.1. Since *n* KANs are used to represent the local orientations of *n* rod segments, each sub‐network is constructed using a single KAN layer followed by a fully connected layer to reduce the overall parameter scale. The number of sampling points on each rod segment is set to 512. To establish the fixed assembling relationship among rod segments connected at a vertex, an orientation hard constraint is imposed on the rod segments. Specifically, for the rod connecting the *i*‐th and (*i*+1)‐th vertices, with global vertex orientations $Q_{v}^{\left(i\right)}$ and $Q_{v}^{\left(i+1\right)}$, the global orientations of the rod at its starting and ending points are defined as $Q_{r,0}^{\left(i\right)}=Q_{v}^{\left(i\right)}\circ A_{i}^{\left(i\right)}$ and $Q_{r,L}^{\left(i\right)}=Q_{v}^{\left(i+1\right)}\circ A_{i}^{\left(i+1\right)}$, respectively, where the symbol “∘” represents the Hamilton quaternion product. Accordingly, the final output of the *i*‐th rod's orientation field $q_{r}^{\left(i\right)}$ is expressed as

(1) $$\begin{array}{ccc} q_{r}^{\left(i\right)}\left(s^{\left(i\right)}\right) & = & \mathrm{Normalize}\left[Q_{r,0}^{\left(i\right)}\left(1-s^{\left(i\right)}\right)+Q_{r,L}^{\left(i\right)}s^{\left(i\right)}\right.\\ & & +\;\left.s^{\left(i\right)}\left(1-s^{\left(i\right)}\right)NN^{\left(i\right)}\left(s^{\left(i\right)};\theta^{\left(i\right)}\right)\right] \end{array}$$

As formulated in Equation (1), the orientations at the two endpoints of each rod segment are directly prescribed by the vertex orientations $Q_{v}^{\left(i\right)}$ and $Q_{v}^{\left(i+1\right)}$, while the KAN sub‐network provides the internal temporary quaternion components (Figure 2A‐iii). The term *s* ^(^ *^i^* ^)^(1−*s* ^(^ *^i^* ^)^) ensures that the output of the network vanishes at both boundaries, thereby guaranteeing the orientation of the rod endpoint aligns with the vertex assembling posture, and continuity. A normalization operation is further applied to ensure the physical validity of the unit quaternion.

In our framework, each rod segment is modeled as an inextensible and unshearable Kirchhoff rod, which means that we only need to solve the local rotation along the arc length to reconstruct the rod configuration. For a rod segment connecting the *i*‐th vertex and the *j*‐th vertex, the rod's starting point position vector $p_{r,0}^{\left(i\right)}$ is taken to be the same as the vertex position vector $p_{v}^{\left(i\right)}$. In other words, the rod starts from the vertex, and this starting point is fixed as a hard constraint (Figure 2A‐iii). According to Kirchhoff rod kinematics, i.e., **p′** = **e**_3_, the global position at any point *s* ^(^ *^i^* ^)^ along the rod can be determined by integrating the tangent field (see details of Kirchhoff rod model in Section S1 of Supporting Information). In this way, once the orientation field is known, the full rod configuration and the segmented ring can be reconstructed (see Section S2 of the Supporting Information for details). The endpoint of the rod segment is obtained as $p_{r,L}^{\left(i\right)}$, and the rod endpoint closure condition $L_{C}^{\left(i\right)}$ is defined as

(2) $$L_{C}^{\left(i\right)}={\left\|\Delta p^{\left(i\right)}\right\|}_{2}^{2}$$

where $\Delta p^{\left(i\right)}=p_{v}^{\left(j\right)}-p_{r,L}^{\left(i\right)}$ is the rod endpoint position deviation vector, and ${∥\cdot ∥}_{2}^{2}$ denotes the square Euclidean norm.

Moreover, in the absence of external forces, self‐contact, and gravity, the total potential energy of the rod system is equal to its strain energy. Although the ring may undergo large overall deformation during shape morphing, the local strains in the rod segments remain small due to their slender geometry. Therefore, a linear elastic constitutive relation is adopted to describe the local deformation of the rod segments. According to KRT, the strain energy $L_{U}^{\left(i\right)}$ of the *i*‐th rod segment is written as

(3) $$\begin{array}{ccc} L_{U}^{\left(i\right)} & = & \frac{1}{2}\int \left[EI_{1}{\left(\kappa_{1}^{\left(i\right)}-\kappa_{n1}^{\left(i\right)}\right)}^{2}+EI_{2}{\left(\kappa_{2}^{\left(i\right)}-\kappa_{n2}^{\left(i\right)}\right)}^{2}\right.\\ & & +\left.GJ{\left(\kappa_{3}^{\left(i\right)}-\kappa_{n3}^{\left(i\right)}\right)}^{2}\right]ds^{\left(i\right)} \end{array}$$

where *κ*_1_, *κ*_2_, and *κ*_3_ are the curvatures of the rod segment about the local frame **e**_1_, **e**_2_, and **e**_3_, respectively, and *κ*_n1_, *κ*_n2_, and *κ*_n3_ are the corresponding in‐plane natural curvature, out‐of‐plane natural curvature, and twisting natural curvature. Moreover, *E* is the Young's modulus, *G* is the shear modulus, *I*_1_ and *I*_2_ denote the moments of inertia along the width and height directions, respectively, and *J* is the torsional constant. Combined with the rod endpoint closure condition $L_{C}^{\left(i\right)}$, the physics constraints of the KRT‐PINN framework can be obtained (Figure 2B). Therefore, the total loss function for forward prediction of the stable states of segmented rings is defined as

(4) $$L_{\mathrm{for}}=\sum_{i=1}^{n}\left(L_{U}^{\left(i\right)}+\lambda_{C}L_{C}^{\left(i\right)}\right)$$

where *λ*_C_ is a constant penalty coefficient for enforcing the closure condition.

Considering the *NN* ^(^ *^i^* ^)^ as trial functions of the configurations of rod segments, the training process of the neural network is a variation of the total potential energy under closure conditions. The trained networks represent stable configurations corresponding to the local minimum of the total potential energy. To avoid convergence to unstable equilibrium branches, the optimization alternates between Adam (Adaptive Moment Estimation) and SSBFGS (Self‐scaled Broyden‐Fletcher‐Goldfarb‐Shanno), where Adam is used for efficient exploration in the early stage of training, and SSBFGS is adopted for accurate final convergence (see details in Section 4 for Methods and Experiments) [40, 41]. The training process is terminated when the variation of the total loss between successive iterations becomes smaller than the predefined threshold *G_L_*.

In the forward prediction, the natural curvature profile of each rod segment is prescribed (Figure 2C), and the network aims to predict the resulting stable configuration of the segmented ring. In the present study, we consider only the out‐of‐plane natural curvature *κ*_n2_ of the rod segments (i.e., *κ*_n1_ = *κ*_n3_ = 0), which induces bending moments that deform the initially planar 2D segmented ring into a 3D stable configuration. A constant value of *κ*_n2_ corresponds to a rod with uniform natural curvature, whereas a varying *κ*_n2_(*s*) describes a nonuniform natural curvature distribution along the arc length. The stable configuration associated with the prescribed natural curvature profile is then obtained by optimizing the parameters *θ* ^(^ *^i^* ^)^ of all sub‐networks, together with the position vector $p_{v}^{\left(i\right)}$ and orientation quaternions $Q_{v}^{\left(i\right)}$ of all vertices to minimize the total loss function, i.e., Equation (4). It should be noted that a 2D segmented ring with out‐of‐plane natural curvature may have multiple stable states. In such cases, different stable configurations can be identified through randomized initializations of $p_{v}^{\left(i\right)}$ and $Q_{v}^{\left(i\right)}$.

Building upon this forward solver, the inverse design strategy is also formulated as a multi‐objective optimization problem. In this work, the objective of the inverse design is to determine the natural curvature profile of the rod segments such that the resulting stable ring configuration can be confined within a prescribed spatial domain or conform to a prescribed curved surface, as illustrated in Figure 2D. To achieve this, an additional shape‐matching loss *L*_shape_ is introduced to quantify the discrepancy between the predicted ring stable configuration and the prescribed target surface or domain. The detailed algorithms for *L*_shape_ are provided in Section S3 of the Supporting Information. Accordingly, the total loss function for the inverse design of stable configurations of segmented rings is defined as

(5) $$L_{\mathrm{inv}}=\sum_{i=1}^{n}\left(L_{U}^{\left(i\right)}+\lambda_{C}L_{C}^{\left(i\right)}\right)+\lambda_{S}L_{\mathrm{shape}}$$

where *λ*_S_ is the penalty coefficient associated with shape matching. Note that in the inverse design, the out‐of‐plane natural curvature is allowed to vary along the arc length, i.e., $\kappa_{n2}^{\left(i\right)}=f^{\left(i\right)}\left(s^{\left(i\right)}\right)$, and is set as a trainable quantity. By simultaneously optimizing the network parameters *θ* ^(^ *^i^* ^)^, the position vectors $p_{v}^{\left(i\right)}$ and orientation quaternions $Q_{v}^{\left(i\right)}$ of all vertices, and the natural curvature profile $\kappa_{n2}^{\left(i\right)}$ to minimize the total loss function, i.e., Equation (5), the optimal natural curvature distribution of the rod segments can be identified by the KRT‐PINN framework, thereby endowing the segmented ring with the ‘material intelligence’ required to achieve programmed configuration design. Note that the inverse design solution of ring origami for a prescribed target shape is generally non‐unique, since different natural curvature distributions may lead to similar final target configurations. In the present work, the inverse design is formulated as an optimization problem that searches for one natural curvature profile whose forward‐predicted configuration matches the prescribed target. Therefore, the obtained design should be interpreted as one feasible solution, rather than the unique solution of the inverse problem. To reduce the degree of solution non‐uniqueness, improve convergence efficiency, and simplify fabrication, symmetry constraints can be imposed on the natural curvature profiles during inverse design, for example, by considering all rod segments having the same natural curvature profile.

Additionally, within the KRT‐PINN framework, the static equilibrium behavior of ring origami is governed primarily by the prescribed natural curvature, ring geometry, and relative bending/torsional stiffness ratios [11]. For isotropic materials, these stiffness ratios depend only on the cross‐sectional geometry and Poisson's ratio. As a result, changing the elastic modulus primarily scales the total elastic energy and force level, but does not affect the predicted equilibrium state, as long as the structure remains within the linear elastic Kirchhoff rod regime. For anisotropic materials, however, the bending/torsional stiffness ratios also depend on the material properties. In this case, the model can be extended by incorporating anisotropic constitutive relations into the strain energy formulation. Moreover, all design variables in the framework are nondimensionalized, allowing the framework to be scaled to different geometric sizes and application scenarios, provided that the linear elastic, inextensible, and unshearable assumptions of KRT remain valid.

### 2.2 Forward Prediction of 2D Segmented Rings

**FIGURE 3:**
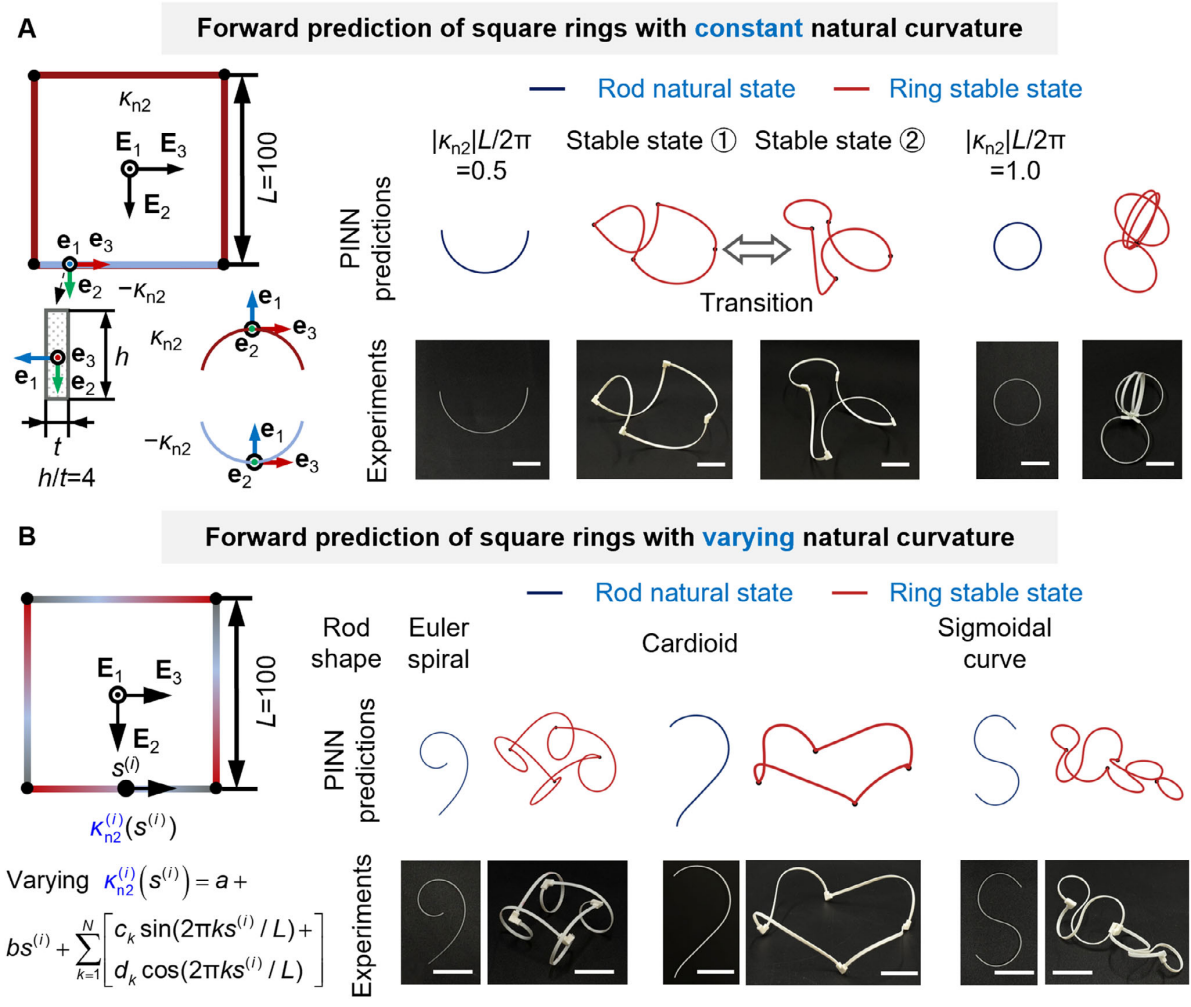
Forward prediction of stable states of square rings with prescribed out‐of‐plane natural curvature. **A**) Forward prediction of square rings with constant natural curvature: (Left) Schematic of a square ring composed of three rod segments with positive constant natural curvature *κ*_n2_ and one rod segment with negative constant natural curvature −*κ*_n2_, where rod segments with positive and negative natural curvature are marked in red and blue, respectively. (Right) Stable states of square rings with dimensionless natural curvatures |*κ*_n2_|*L*/2π = 0.5 and 1.0, predicted by the KRT‐PINN framework and validated experimentally. **B**) Forward prediction of square rings with varying natural curvature. (Left) Schematic of a square ring composed of rod segments sharing the same varying natural curvature. The natural curvature profile $\kappa_{n2}^{\left(i\right)}\left(s^{\left(i\right)}\right)$ is defined by a Fourier series parameterization with linear terms. (Right) Stable states of square rings composed of rod segments with different prescribed natural shapes, including an Euler spiral, a cardioid, and a sigmoidal curve, predicted by the KRT‐PINN framework and validated experimentally. Scale bars: 20 mm.

To demonstrate the accuracy and reliability of the proposed KRT‐PINN framework, we first employ it to predict the stable states of 2D segmented rings with prescribed out‐of‐plane natural curvature. Square rings consisting of rod segments with constant or varying natural curvature are considered as representative examples. The side length and cross‐sectional height‐to‐width ratio of the square ring are fixed at *L* = 100 mm and *h*/*t* = 1/4, respectively. As shown in Figure 3A, a square ring composed of three rod segments with constant positive natural curvature *κ*_n2_ (indicated by red) and one rod segment with constant negative natural curvature −*κ*_n2_ (indicated by blue) is studied. Note that a positive natural curvature induces a moment that bends the rod segment upward, toward the **e**_1_‐direction, whereas a negative natural curvature induces a moment that bends the rod segment downward, opposite to the **e**_1_‐direction. When the dimensionless natural curvature |*κ*_n2_|*L*/2π = 0.5, two distinct 3D stable states of the square ring are identified by the KRT‐PINN framework through randomly initializing the ring configuration, as illustrated by stable states ① and ② in Figure 3A. The two predicted stable configurations are validated by experiments (see details in Section 4 for Methods and Experiments), showing good consistency. By applying external bending loads, the two stable states can transition between each other through snapping. In contrast, when |*κ*_n2_|*L*/2π increases to 1, the KRT‐PINN framework identifies only one stable state, in which the square ring reaches equilibrium in a 3D figure “8” configuration. In this case, the edge curvature of the stable configuration is close to the natural curvature of the rod segments, indicating a nearly zero‐energy stable state.

Figure 3B presents the stable states of square rings consisting of rod segments with the same varying natural curvature, predicted by the KRT‐PINN framework. The varying natural curvature profile of each rod segment is defined as a function of its arc length *s* ^(^ *^i^* ^)^ by a Fourier series parameterization with additional linear terms:

(6) $$\kappa_{n2}^{\left(i\right)}\left(s^{\left(i\right)}\right)=a+bs^{\left(i\right)}+\sum_{k=1}^{N}\left[c_{k}\sin\left(2\pi ks^{\left(i\right)}/L\right)+d_{k}\cos\left(2\pi ks^{\left(i\right)}/L\right)\right]$$

where the coefficients *a*, *b*, *c_k_*, and *d_k_* are constants determined by the prescribed natural curvature profile. Using this parameterization, three typical types of varying natural curvature distributions are considered for the square ring, including an Euler spiral, a cardioid, and a sigmoidal curve (Figure 3B). The corresponding coefficients in Equation (6) are provided in Table S1 of the Supporting Information. As shown in Figure 3B, for all three cases, the KRT‐PINN framework successfully predicts the complex 3D stable configurations of square rings with varying natural curvature, in good agreement with the experiments. These results demonstrate that the KRT‐PINN framework is capable of handling ring origami composed of rod segments with general natural curvature profiles.

It should be noted that although only square rings with constant or varying natural curvature are presented here, the proposed KRT‐PINN framework also applies to other ring geometries, regardless of the natural curvature profile, cross‐sectional aspect ratio, or number of rod segments. Additionally, unlike many iterative numerical methods for nonlinear systems, whose convergence may depend strongly on the initial values, the present framework is largely insensitive to initialization and consistently converges to stable states from randomized initial ring configurations. The proposed KRT‐PINN framework also exhibits higher computational efficiency than the traditional finite element method (FEM), particularly for rings with varying natural curvatures. As detailed in Section S4.1 and Table S2 of the Supporting Information, under the same discrete scale of the rod axial direction settings, the proposed framework reduces computational time by 30%–60% compared with shell‐element‐based FEM for square rings with varying natural curvature profiles. Furthermore, the computational time of the proposed KRT‐PINN framework depends on the number of rod segments. A quantitative comparison of the computational efficiency of 2D rings and 3D rod systems with different numbers of rod segments is provided in Section S4.2 and Table S3 of the Supporting Information. In general, within the same category of rod systems (2D or 3D), the forward prediction time tends to increase as the number of rod segments increases, since more trainable parameters and nodal variables are introduced. Across different categories of rod systems, however, the overall computational time is also influenced by the optimization complexity associated with the system topology. This robust and generalized framework, together with its reliable convergence behavior and high computational efficiency, lays the foundation for the subsequent inverse design of 2D segmented rings to achieve the target stable configurations.

### 2.3 Inverse Design of 2D Segmented Rings Confined Within Target Spatial Domains

**FIGURE 4:**
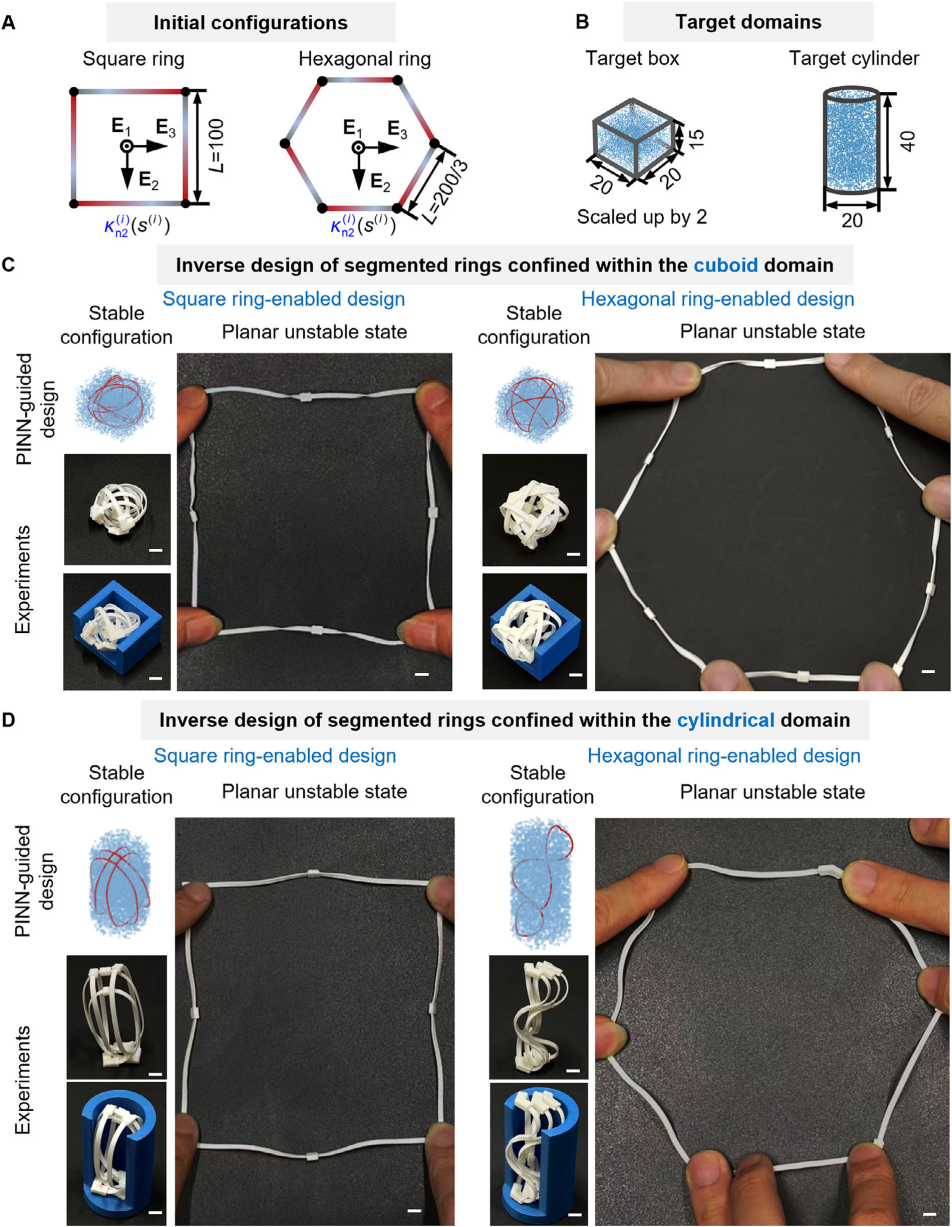
Inverse design of segmented rings confined within prescribed spatial domains. (A) Initial planar configurations of square and hexagonal rings composed of rod segments with varying natural curvature profiles. The two rings have the same total length of 400 mm. (B) Target spatial confinement domains: a cuboid box (20 × 20 × 15 mm) and a slender cylinder with a diameter of 20 mm and a length of 40 mm. The schematics of the target box is scaled up by 2 with respect to the initial ring configurations. (C) Inverse design of segmented rings confined within the cuboid domain: Stable configurations of the (Left) square ring and (Right) hexagonal ring predicted by the KRT‐PINN framework and validated experimentally. (D) Inverse design of segmented rings confined within the cylindrical domain: Stable configurations of the (Left) square ring and (Right) hexagonal ring predicted by the KRT‐PINN framework and validated experimentally. In panels (C,D), experimental images of the initial planar unstable states are also presented. Scale bars: 5 mm.

Having verified the accuracy and reliability of the proposed KRT‐PINN framework for forward prediction, we next employ the framework for inverse design of segmented rings to achieve stable configurations that can be confined within target spatial domains. Such an inverse design problem is commonly encountered in the design of foldable structures for aerospace applications, where the structures are required to be compactly stowed for efficient storage and transportation [2, 42]. To demonstrate this capability, we consider two ring topologies: a square ring and a hexagonal ring, as shown in Figure 4A. The two rings have the same total length of 400 mm, and all rod segments in each ring have identical lengths. As mentioned previously, to reduce the degree of solution non‐uniqueness, improve convergence efficiency, and simplify fabrication, all rod segments are assumed to share an identical natural curvature profile in the inverse design. Two target spatial domains are considered: a cuboid box with dimensions of 20 × 20 × 15 mm and a slender cylinder with a diameter of 20 mm and a length of 40 mm (Figure 4B).

Figure 4C shows the inverse design results for square and hexagonal rings confined within the prescribed cuboid domain, obtained by optimizing the natural curvature distribution of the rod segments using the KRT‐PINN framework. The corresponding optimized natural curvature profiles are provided in Figure S1 of the Supporting Information. As indicated by the point cloud, the predicted stable configurations of both rings are successfully confined within the target domain, demonstrating the proposed framework's applicability to different ring topologies. Experimental results further validate these predictions. With the optimized natural curvature distributions, both the planar square and hexagonal rings lose stability and spontaneously snap, without external constraints, into compact 3D stable configurations that are confined within the prescribed cuboid domain. These 3D configurations occupy only approximately 4% of the initial planar area, highlighting the excellent packing capability of the segmented rings. Note that, in the experiments, the finite physical volume of the rod segments and joints makes the final stable configurations slightly larger than those predicted by the KRT‐PINN framework. This effect is more pronounced in the hexagonal ring because it contains more rod segments and joints.

Inverse design of square and hexagonal rings confined within the prescribed cylindrical domain is presented in Figure 4D. As predicted by the KRT‐PINN framework and validated by experiments, the square and hexagonal rings, assembled by rod segments with the optimized natural curvature profiles (Figure S1), become unstable from their initial planar state. Once the external constraints are removed, they spontaneously snap into axially elongated 3D configurations that can be confined within the slender cylindrical domain, thereby enabling highly efficient packing with an areal packing ratio of approximately 3%. For the hexagonal ring, the predicted stable configuration exhibits a three‐layer overlapping arrangement. However, as noted above, due to the finite thickness of the printed rods and joints, the experimental configuration does not show perfect overlap and occupies a slightly larger volume. These finite‐size geometric effects are not explicitly considered in the present centerline‐based formulation. Incorporating them into the model would require a contact‐aware treatment with additional non‐penetration constraints, which may further improve the quantitative accuracy of the inverse design results. These demonstrations also confirm that the KRT‐PINN framework can be used for the inverse design of segmented rings across different target spatial domains and initial topologies.

### 2.4 Inverse Design of 2D Segmented Rings Conforming to Target Surfaces

**FIGURE 5:**
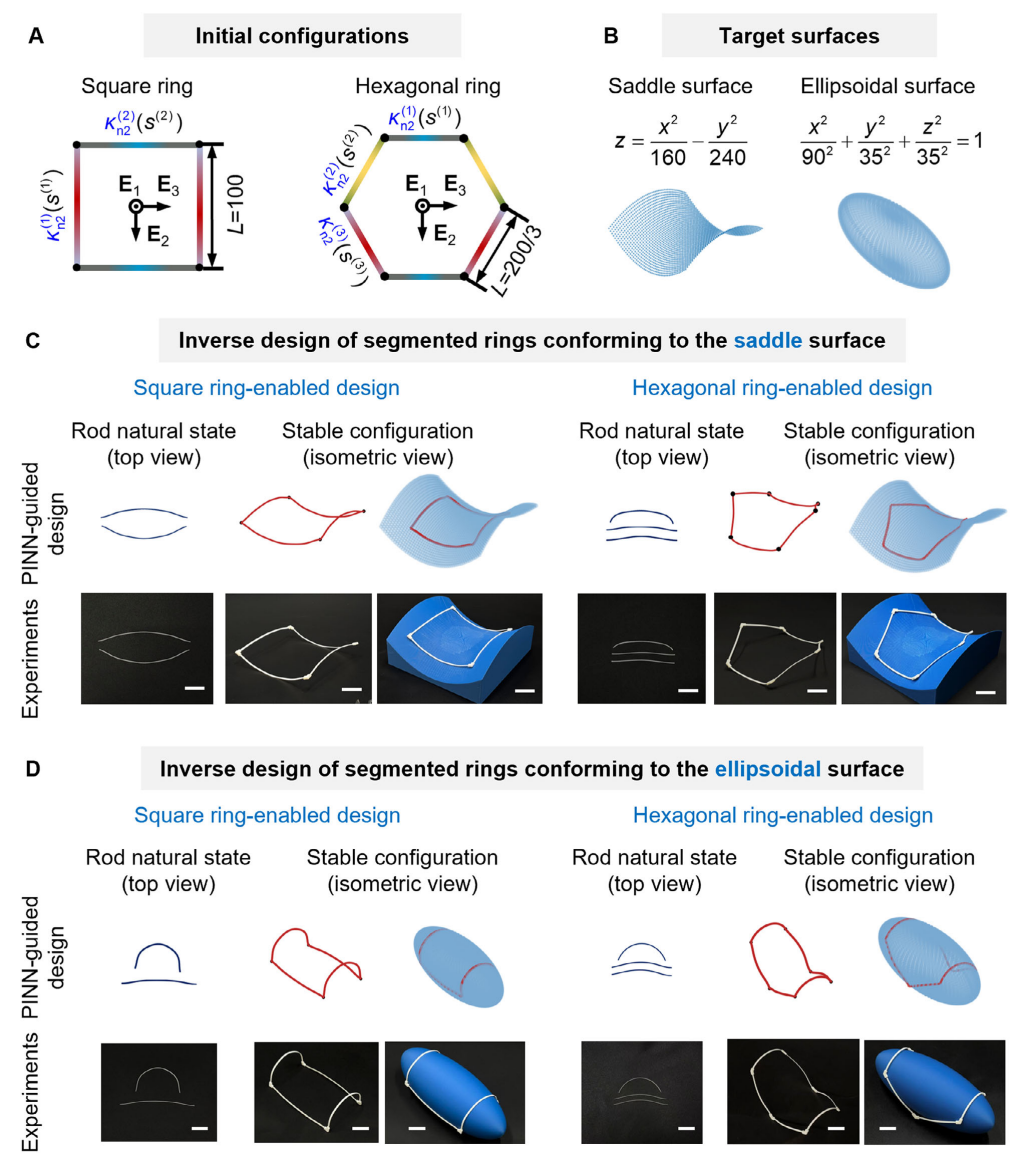
Inverse design of segmented rings conforming to prescribed curved surfaces. (A) Initial planar configurations of square and hexagonal rings with the same total length of 400 mm. To better conform to the target surfaces, the square and hexagonal rings use two and three groups of varying natural curvatures for their rod segments, respectively, as indicated by the gradient lines in the schematics. (B) Target curved surfaces: a saddle surface with negative Gaussian curvature and an ellipsoidal surface with positive Gaussian curvature. (C) Inverse design of segmented rings conforming to the saddle surface: Natural states of the rod segments and stable configurations of the (Left) square ring and (Right) hexagonal ring, predicted by the KRT‐PINN framework and validated experimentally. (D) Inverse design of segmented rings conforming to the ellipsoidal surface: Natural states of the rod segments and stable configurations of the (Left) square ring and (Right) hexagonal ring, predicted by the KRT‐PINN framework and validated experimentally. Scale bars: 20 mm.

In engineering applications, some morphing structures are required to attain specific shapes to realize specific functions. For example, space antennas often adopt parabolic geometries to improve signal focusing and reflection [24]. In this subsection, we further demonstrate that the KRT‐PINN framework can be used for the inverse design of segmented rings to achieve stable configurations that conform to target curved surfaces. Consistent with Section 2.3, we use both square and hexagonal rings with the same total length to enable the design. As shown in Figure 5A, to reduce the degree of solution non‐uniqueness, simplify fabrication, as well as better conform to the target surfaces, opposite rod segments in the square ring are assigned identical natural curvature profiles, while the rod segments in the hexagonal ring are divided into three pairs of opposite rod segments, with each pair sharing the same natural curvature profile. Two representative target curved surfaces are considered: a saddle surface with negative Gaussian curvature and an ellipsoidal surface with positive Gaussian curvature (Figure 5B). The saddle surface has a hyperbolic geometry and is described by *z* = *x* ^2^/160 − *y* ^2^/240, where *x*, *y* ∈ [−60, 60]. In contrast, the ellipsoidal surface represents a doubly curved geometry with positive Gaussian curvature and is defined by *x* ^2^/90^2^ + *y* ^2^/35^2^ + *z* ^2^/35^2^ = 1.

Using the KRT‐PINN framework, the optimized natural curvature profiles (Figure S2) and the resulting stable configurations of the square and hexagonal rings are determined. As shown in Figure 5C, the two optimized natural curvatures induce alternating bending directions in the rod segments of the square ring, enabling it to conform to the prescribed saddle surface. For the hexagonal ring, one relatively large natural curvature and two smaller natural curvatures are assigned to its three pairs of rod segments, allowing the ring to conform to the target saddle surface. The predictions are further validated experimentally, showing good agreement. Figure 5D shows the inverse design of square and hexagonal rings conforming to the prescribed ellipsoidal surface. In both cases, one pair of opposite rod segments is assigned a relatively large natural curvature to accommodate the larger curvature along the circumferential direction of the ellipsoidal surface, whereas the remaining rod segments are assigned relatively small natural curvatures to fit the smaller curvature along the longitudinal direction. With these optimized natural curvature profiles (Figure S3), the initial planar segmented rings deform into 3D equilibrium configurations that conform to the target surface with positive Gaussian curvature. The predicted stable configurations agree well with the experimental results for both square and hexagonal rings. These results further demonstrate that the proposed KRT‐PINN framework can program segmented rings of different geometries to conform to surfaces with qualitatively different curvature characteristics.

Note that in the inverse design examples presented in Figures 4 and 5, the predicted configurations are all monostable. Thus, when the structure is perturbed away from the designed configuration, it tends to spontaneously return to the same minimum‐energy stable state once the perturbation is removed. The energy landscape of the square ring conforming to the saddle surface under external perturbations, imposed as a pair of rotations at two opposite edges, is shown in Figure S4 of the Supporting Information. The energy of the stable state is much lower than that of the perturbed configurations, indicating that the obtained designs are physically stable against small perturbations. For potentially multistable designs, transitions to other stable states can occur only when the applied perturbation provides sufficient energy to overcome the energy barrier between stable configurations. Such transitions are reversible, meaning that the ring can return to its original stable configuration when an appropriate reverse perturbation is applied. In this case, the stability of a designed configuration against perturbation is governed by its local energy minimum and the corresponding energy barriers separating it from other possible stable states.

### 2.5 Forward Prediction of 3D Rod Systems

**FIGURE 6:**
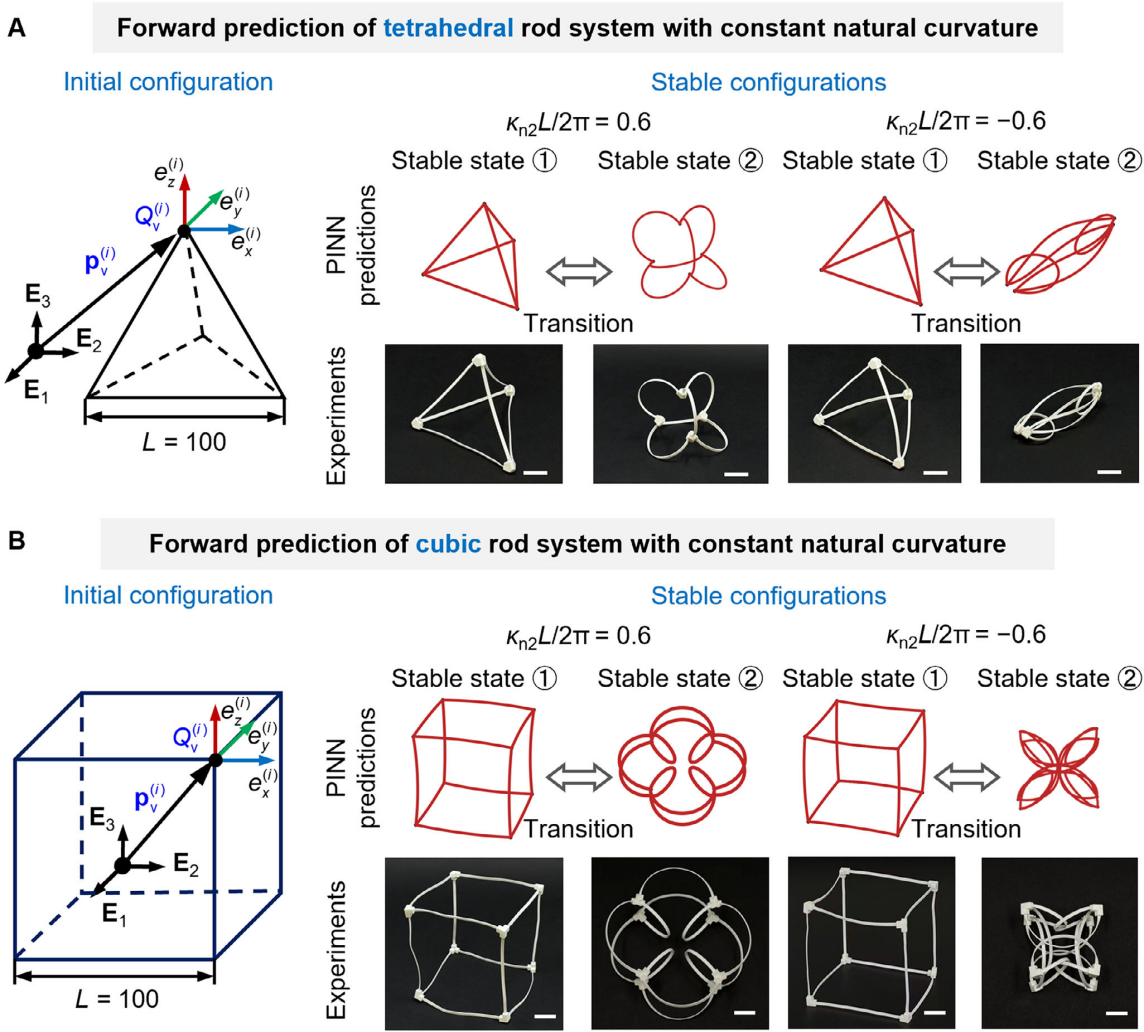
Forward prediction of stable states of 3D rod systems based on the KRT‐PINN framework. (A) Tetrahedral rod system with constant natural curvature: (Left) Schematic of the initial configuration composed of six rod segments with the same length *L* = 100 mm and the same natural curvature *κ*_n2_. At each vertex, an orientation quaternion $Q_{v}^{\left(i\right)}$ is introduced to describe the joint orientation with respect to the global frame, and a position vector $p_{v}^{\left(i\right)}$ is defined to specify the vertex position. (Right) Stable configurations of the tetrahedral rod system with dimensionless natural curvature *κ*_n2_ *L*/2π = 0.6 and −0.6, predicted by the KRT‐PINN framework and validated experimentally. Note that the experimental configuration of stable state ② corresponding to *κ*_n2_ *L*/2π *=* −0.6 is obtained by allowing edge penetration. (B) Cubic rod system with constant natural curvature: (Left) Schematic of the initial configuration composed of twelve rod segments with the same length *L* = 100 mm and the same natural curvature *κ*_n2_. (Right) Stable configurations of the cubic rod system with dimensionless natural curvature *κ*_n2_ *L*/2π = 0.6 and −0.6, predicted by the KRT‐PINN framework and validated experimentally. Scale bars: 20 mm.

Finally, to further demonstrate the generality and robustness of the developed KRT‐PINN framework, we extend it from 2D rings to 3D rod systems, which are likewise assembled from rod segments with natural curvature. In this case, geometric compatibility and force–moment balance in three dimensions introduce stronger geometric constraints and richer multistable behavior. Here, we focus on the forward prediction of stable configurations in 3D rod systems composed of rod segments with prescribed constant natural curvature. Two representative 3D rod systems are considered: a tetrahedral rod system with six rod segments and a cubic rod system with twelve rod segments, as shown in Figure 6A,B.

Figure 6A presents the stable states of a tetrahedral rod system predicted by the KRT‐PINN framework. All rod segments in the system have the same length *L* = 100 mm and the same prescribed constant natural curvature. When the dimensionless natural curvature is *κ*_n2_ *L*/2π = 0.6, the framework identifies two stable states, labeled as stable state ① and stable state ②. Stable state ① preserves the initial tetrahedral topology, whereas stable state ② exhibits a distinct configuration in which each rod segment has nearly uniform curvature. The two stable states are well validated by experiments, which further show that they can transition into each other through collective vertex inversion under external stimuli. When the dimensionless natural curvature is changed to *κ*_n2_ *L*/2π = −0.6, which is achieved experimentally by reversing the rod orientation during assembly, the rod system still exhibits two stable states, and the initial tetrahedron configuration remains stable (i.e., stable state ①). However, stable state ② exhibits a new configuration distinct from that for *κ*_n2_ *L*/2π = 0.6, which adopts a more compact, spindle‐like morphology. In this case, experiments show that the transition between the two stable states requires edge penetration. This indicates that the rod orientation during assembly has a distinct influence on the stable configurations of 3D rod systems.

The stable states of a cubic rod system with prescribed constant natural curvature are shown in Figure 6B. The system is assembled from rod segments with the same length of *L* = 100 mm. As predicted by the KRT‐PINN framework and validated experimentally, the cubic rod system also exhibits multistability when *κ*_n2_ *L*/2π = 0.6 and −0.6. For both values of natural curvature, stable state ① largely retains the initial cubic configuration, with only slight edge bending induced by the natural curvature, while stable state ② exhibits a distinct configuration in which the rod segments are highly curved. Similar to the tetrahedral rod system, the two stable states can transition into each other under external stimuli. Moreover, stable state ② for *κ*_n2_ *L*/2π = −0.6 is more compact than that for *κ*_n2_ *L*/2π = 0.6.

The results in Figure 6 demonstrate that the KRT‐PINN framework is not limited to 2D rings but can also predict the stable states of 3D rod systems. It can therefore serve as a powerful tool for guiding the design of diverse elastic rod systems involving complex geometric constraints and rich nonlinear behavior. For the inverse design of 3D rod systems, the framework can identify stable equilibrium configurations that satisfy the prescribed geometric target. However, because contact between rod segments is not explicitly considered in the current framework, the deformation pathway from the initial configuration to the predicted stable configuration may involve edge penetration or self‐intersection. Consequently, the designed structures, especially densely packed configurations, may not be experimentally realizable, since contact between rod segments can block the required deformation pathways. Incorporating contact mechanics would therefore be an important extension of the present framework. Specifically, contact effects could be introduced through non‐penetration constraints or contact‐penalty terms in the loss function. Such an extension would improve the physical feasibility and practical applicability of the framework for densely packed 3D rod systems and make the inverse design results more reliable for compact, contact‐dominated configurations. In addition, recent studies have demonstrated the success of data‐driven and physics‐informed graph neural networks (GNNs) in modeling nonlinear mechanics problems, including rod dynamics [43, 44, 45]. Exploring graph‐based learning paradigms for ring origami and other rod systems would be an interesting direction for future research.

## 3 Conclusions

In summary, we have developed a KRT‐PINN framework for the forward prediction and inverse design of ring origami composed of rod segments with natural curvature. By incorporating Kirchhoff rod theory into a physics‐informed neural network, the framework formulates an energy‐variational optimization problem to minimize the total potential energy of the ring, thereby enabling forward prediction of stable states for prescribed natural curvature profiles. By introducing an additional shape‐matching loss, the framework can determine the natural curvature profile required for segmented rings to achieve target stable configurations. To demonstrate the accuracy and reliability of the proposed framework, forward prediction of stable states of square rings with prescribed constant or varying natural curvature, as well as inverse design of square and hexagonal rings to realize stable configurations that can be confined within a target spatial domain or conform to a target curved surface, are investigated. The results show good agreement with experimental validations. The generality and robustness of the developed framework are further demonstrated through its extension to 3D rod systems, where it successfully predicts the multiple stable states of tetrahedra and cubes assembled from rod segments with natural curvature. These results establish the KRT‐PINN framework as a powerful tool for the programmable design of elastic rod systems exemplified by ring origami, with great potential for functional applications requiring shape‐morphing structures with simple geometries, high packing capability, and prescribed stable configurations.

## 4 Methods and Experiments

### 4.1 Training Setup for the KRT‐PINN Framework

In the forward prediction training of the KRT‐PINN framework, the initial training stage is implemented by the Adam optimizer with 500 epochs. The network parameters are optimized with an initial learning rate of 2 × 10^−4^. To ensure stable convergence, a step‐based learning rate decay strategy is implemented, where the learning rate is reduced by a factor of 0.5 every 200 epochs. The main optimization stage is carried out using an SSBFGS algorithm adapted from Reference [41] and implemented in PyTorch. The optimizer is configured with a relatively large learning rate (0.8) together with a strong Wolfe line search strategy to ensure stable convergence. The maximum number of internal iterations per step is set to 3, while strict convergence tolerances are imposed on both the gradient norm (10^−8^) and parameter updates (10^−12^). During each iteration, the total loss is defined as the sum of the potential energy and a weighted closure constraint term. The penalty coefficient of the closure constraint is set to a maximum value of 300. To mitigate stagnation in local minima, an auxiliary Adam optimizer is introduced as a perturbation mechanism. When the loss fails to improve beyond a prescribed threshold for a given number of iterations (patience = 10), the SSBFGS optimization is temporarily interrupted. A small number of Adam steps (20 iterations) with a reduced learning rate (2 × 10^−4^) are then performed to perturb the solution and promote exploration of the loss landscape. After this perturbation phase, the SSBFGS optimizer is reinitialized and resumed from the updated parameters.

The convergence behavior, prediction accuracy, training stability, and parameter efficiency of the conventional MLP‐based PINN and the KAN‐based PINN are compared in Figures S5–S7 and Table S4 in the Supporting Information. In the comparison, both networks are trained using the same geometric settings, loss functions, sampling strategy, and optimization procedure, with details provided in Section S4.3 of the Supporting Information. The results show that, for the ring origami problems considered in this work, the KAN‐based PINN exhibits better convergence behavior, higher prediction accuracy, improved training stability, and greater parameter efficiency than the MLP‐based PINN.

For the inverse design training of the KRT‐PINN framework, a hybrid optimization strategy combining Adam and SSBFGS is also employed to mitigate stagnation in local minima. The parameter setting is the same as that of the forward prediction. In particular, the inverse design consists of two stages. In the first stage, the loss function includes a shape‐matching penalty that drives the ring configuration toward the prescribed target shape. To avoid falling into local optima, the penalty coefficient of the shape‐matching loss is periodically increased from 0 to 300 over 300 epochs. In the second stage, the penalty coefficient is set to zero for an additional 200 epochs. During this stage, the loss function includes only the elastic energy loss, which drives the ring configuration obtained in the first stage toward a true equilibrium configuration. A total of 1400 epochs are used to solve each inverse design problem, including four cycles of the penalty‐enforcing and penalty‐releasing procedure for the shape‐matching loss. In the final converged state, a spring‐back is observed, characterized by a small deviation between the shape‐matching penalty‐enforced configuration obtained at the end of the first stage and the final released equilibrium configuration obtained in the second stage. A quantitative comparison of the configurations before and after releasing the shape‐matching loss for the inverse design of square and hexagonal rings conforming to a saddle is illustrated in Figure S8 in the Supporting Information. In both cases, the configuration deviation is negligibly small, with maximum deviations of 1.267 and 1.651 mm for square and hexagonal rings, respectively, indicating that the final stable configurations still agree well with the target geometry. At present, this residual spring‐back is hard to completely eliminate, as the sensitivity of the spring‐back amplitude with respect to the parameters in the trial function of the rod natural curvature cannot be evaluated explicitly.

All models are implemented in PyTorch (version 2.3.1) with CUDA 12.1 support, and the computations are performed on a system equipped with an Intel i9‐13950HX CPU, an NVIDIA RTX 4060 GPU, and 32 GB of RAM.

### 4.2 Experimental Fabrication of the Rings

The experimental rings and 3D rod systems are fabricated by assembling multiple rod segments through rigid joints. The rod segments and joints are 3D printed using a Fused Deposition Modeling (FDM) printer with polylactic acid (PLA) as the printing material. The Young's modulus and Poisson's ratio of the material are *E* = 2.1 GPa and *ν* = 0.33, respectively. All rod segments have a rectangular cross‐section with height *h* = 0.5 mm and width *t* = 2 mm, while the lengths vary as specified in the main text. It should be noted that the proposed KRT‐PINN framework is not restricted to PLA itself, but can, in principle, be extended to other materials, provided that their mechanical behavior remains within the validity range of the current Kirchhoff rod model. For more compliant materials, additional effects may become important in practice, including shear/stretch deformation, viscoelasticity, material nonlinearity, and gravity‐induced sagging. These effects are not explicitly included in the present formulation and may reduce the quantitative prediction accuracy if such materials are used without further model extension.

## Author Contributions

**Luyuan Ning**: methodology, writing – original draft, investigation, software, validation, formal analysis, data curation. **Lu Lu**: writing – review and editing, validation, methodology, formal analysis. Sophie Leanza: validation, visualization. **Ruike Renee Zhao**: conceptualization, funding acquisition, project administration, resources, supervision, writing – review and editing, formal analysis.

## Funding

This work was supported by the National Science Foundation Award CPS‐2201344 and National Science Foundation Career Award CMMI‐2145601.

## Conflicts of Interest

The authors declare no conflicts of interest.

## Supporting information




**Supporting File**: advs76194‐sup‐0001‐SuppMat.pdf.

## Data Availability

The data that support the findings of this study are available from the corresponding author upon reasonable request.
