# Supplementary material for: Physics‐Informed Neural Network‐Enabled Forward Prediction and Inverse Design of Ring Origami
Source: Adv Sci (Weinh). 2026 Jun 22:e76194. Online ahead of print. doi: 10.1002/advs.76194 (PMC13336866; doi:10.1002/advs.76194)
Supplement: Supplementary file 1 — Supporting File: advs76194‐sup‐0001‐SuppMat.pdf. [file ADVS-9999-e76194-s001.pdf]

Supporting Information

Physics-Informed Neural Network-Enabled Forward Prediction and Inverse Design of Ring Origami

Luyuan Ning, *Lu Lu*, *Sophie Leanza*, *Ruike Renee Zhao**

Table of contents

Supplementary Texts S1-S4

Supplementary Figures S1-S8

Supplementary Tables S1-S4

Supplementary texts

S1. Potential energy of an elastic rod based on Kirchhoff rod theory

Consider an elastic rod with length L and a rectangular cross-section with height h and width t. The centerline of the rod is parameterized by the arc length $s \in [0, L]$, whose spatial position is described by the vector $\mathbf{p}(s) = p_1\mathbf{E}_1+p_2\mathbf{E}_2+p_3\mathbf{E}_3$ in the global material frame ($\mathbf{E}_1$, $\mathbf{E}_2$, $\mathbf{E}_3$). To characterize the local orientation of the rod cross-section during deformation, an orthonormal local material frame ($\mathbf{e}_1$, $\mathbf{e}_2$, $\mathbf{e}_3$) is attached to the cross-section centerline, where $\mathbf{e}_1$ is along the height direction, $\mathbf{e}_2$ is along the width direction, and $\mathbf{e}_3$ is aligned with the tangent direction of the centerline, which indicates $\mathbf{p}' = \mathbf{e}_3$, and the prime $(\bullet)'$ denotes differentiation with respect to the arc length coordinate s. The evolution of the local frame along the rod is governed by the Darboux vector $\boldsymbol{\omega}(s)$, defined through $\mathbf{e}_i' = \boldsymbol{\omega} \times \mathbf{e}_i$, where i = 1, 2, 3, and $\boldsymbol{\omega} = \kappa_1\mathbf{e}_1+\kappa_2\mathbf{e}_2+\kappa_3\mathbf{e}_3$. Here, κ_1 and κ_2 represent bending curvatures about the local frame $\mathbf{e}_1$ and $\mathbf{e}_2$, while κ_3 represents the twisting curvature about the local frame $\mathbf{e}_3$.

Since the elastic rod is modeled as an inextensible and unshearable Kirchhoff rod [1], only bending and twisting deformations are considered. To describe these large bending and twisting deformations, the rotation of the rod cross-section, represented by the local material frame with respect to the global material frame, is parameterized by unit quaternions (q_0, q_1, q_2, q_3), which are given by [2, 3]

$$\begin{bmatrix} \mathbf{e}_1 \\ \mathbf{e}_2 \\ \mathbf{e}_3 \end{bmatrix} = 2\begin{bmatrix} q_0^2+q_1^2-\frac{1}{2} & q_1q_2+q_0q_3 & q_1q_3-q_0q_2 \\ q_1q_2-q_0q_3 & q_0^2+q_2^2-\frac{1}{2} & q_2q_3+q_0q_1 \\ q_1q_3+q_0q_2 & q_2q_3-q_0q_1 & q_0^2+q_3^2-\frac{1}{2} \end{bmatrix}\begin{bmatrix} \mathbf{E}_1 \\ \mathbf{E}_2 \\ \mathbf{E}_3 \end{bmatrix}. \tag{S1}$$

Note that $q_0^2+q_1^2+q_2^2+q_3^2=1$.

Substituting $\mathbf{p}' = \mathbf{e}_3$ into Equation (S1) yields

$$p_1' = 2(q_1q_3+q_0q_2),\ \ p_2' = 2(q_2q_3-q_0q_1),\ \ p_3' = 2(q_0^2+q_3^2)-1. \tag{S2}$$

From Equation (S1) together with the kinematic identity $\mathbf{e}_i' = \boldsymbol{\omega} \times \mathbf{e}_i$, it follows that

$$\begin{cases} \kappa_1 = 2(-q_1q_0'+q_0q_1'+q_3q_2'-q_2q_3'), \\ \kappa_2 = 2(-q_2q_0'-q_3q_1'+q_0q_2'+q_1q_3'), \\ \kappa_3 = 2(-q_3q_0'+q_2q_1'-q_1q_2'+q_0q_3'). \end{cases} \tag{S3}$$

In this work, linear constitutive relations are considered, which gives

$$\begin{cases} M_1 = EI_1(\kappa_1 - \kappa_{\mathrm{n}1}), \\ M_2 = EI_2(\kappa_2 - \kappa_{\mathrm{n}2}), \\ M_3 = GJ(\kappa_3 - \kappa_{\mathrm{n}3}), \end{cases} \tag{S4}$$

where $\kappa_{\mathrm{n}1}$ is the natural curvature of the rod segment about local frame $\mathbf{e}_1$, $\kappa_{\mathrm{n}2}$ is the out-of-plane natural curvature about local frame $\mathbf{e}_2$, and $\kappa_{\mathrm{n}3}$ is the twisting natural curvature about the local basis $\mathbf{e}_3$. Moreover, I_1 and I_2 denote the moments of inertia about the width and height directions, respectively, and J is the torsional constant [4],

$$I_1 = \frac{1}{12}ht^3,\ I_2 = \frac{1}{12}h^3t,\ J = \frac{ht^3}{3}\left[1 - \frac{192}{\pi^5}\frac{t}{h}\sum_{k=1}^{\infty}\frac{1}{(2k-1)^5}\tanh\left(\frac{(2k-1)\pi h}{2t}\right)\right]. \tag{S5}$$

In the absence of external forces, self-contact, and gravity, the potential energy L_U of an elastic rod is equal to its strain energy, which is calculated by

$$L_U = \frac{1}{2}\int\left[EI_1\left(\kappa_1 - \kappa_{\mathrm{n}1}\right)^2 + EI_2\left(\kappa_2 - \kappa_{\mathrm{n}2}\right)^2 + GJ\left(\kappa_3 - \kappa_{\mathrm{n}3}\right)^2\right]\mathrm{d}s. \tag{S6}$$

The total potential energy of a segmented ring composed of multiple rod segments is obtained by summing the strain energy of each rod segment.

S2. Ring configuration reconstruction method

From the quaternions, the corresponding rotation matrix can be calculated by

$$\mathbf{R} = \begin{bmatrix} 1-2q_2^2-2q_3^2 & 2q_1q_2-2q_0q_3 & 2q_1q_3+2q_0q_2 \\ 2q_1q_2+2q_0q_3 & 1-2q_1^2-2q_3^2 & 2q_2q_3-2q_0q_1 \\ 2q_1q_3-2q_0q_2 & 2q_2q_3+2q_0q_1 & 1-2q_1^2-2q_2^2 \end{bmatrix}. \tag{S7}$$

Given $\mathbf{R} = [\mathbf{e}_1, \mathbf{e}_2, \mathbf{e}_3]$, and Kirchhoff rod kinematics $\mathbf{p}' = \mathbf{e}_3$, we can obtain the local tangent vector $\mathbf{e}_3$ of the rod as

$$\mathbf{e}_3 = \left[2q_1q_3+2q_0q_2,\ 2q_2q_3-2q_0q_1,\ 1-2q_1^2-2q_2^2\right]^{\mathrm{T}}. \tag{S8}$$

For a rod segment connecting the i-th vertex and the j-th vertex, the rod starting point position vector $\mathbf{p}_{\mathrm{r},0}^{(i)}$ is set to be the same as the vertex position vector $\mathbf{p}_{\mathrm{v}}^{(i)}$. Denoting the i-th rod length L_i, the rod configuration vector can be expressed as

$$\mathbf{p}_{\mathrm{r}}^{i}(s^{(i)}) = \mathbf{p}_{\mathrm{v}}^{(i)} + L_i\int_0^{s^{(i)}}\mathbf{e}_3^{i}(s^{(i)})ds^{(i)}. \tag{S9}$$

After the configurations of all rod segments are reconstructed, the ring configuration $\mathbf{P}_{\mathrm{r}}$ is established by a union set of each rod

$$\mathbf{P}_{\mathrm{r}} = \bigcup_{i=1}^{n}\left\{\mathbf{p}_{\mathrm{r}}^{(i)}(s^{(i)})\mid s^{(i)}\in[0,\ L^{(i)}]\right\}, \tag{S10}$$

where n denotes the number of rod segments.

S3. Shape-matching loss in the inverse design framework

In the inverse design of segmented rings to achieve a target stable configuration, such as being confined within a target spatial domain or conforming to a target curved surface, a shape-matching loss is augmented in the training process by a penalty function method. The ring configuration and target surface are all represented by discrete sampling points.

Shape-matching loss for being confined within a target spatial domain

For the inverse design of a ring confined within a target spatial domain, the ring configuration $\mathbf{P}_\mathrm{r}$ must lie within the target domain. To achieve this, an inequality constraint term is introduced to penalize the points in the ring configuration that are beyond the target domain boundaries. For a prescribed cuboid domain, the cuboid boundaries are defined as

$$\mathbf{p}_{\min} = \left[x_{\min}, y_{\min}, z_{\min}\right]^\mathrm{T}, \ \mathbf{p}_{\max} = \left[x_{\max}, y_{\max}, z_{\max}\right]^\mathrm{T}. \tag{S11}$$

To implement gradient-based optimization, the penalty term is formulated using the $\mathrm{ReLU} = \max(0,\ a)$ function as a differentiable surrogate for inequality constraints. Therefore, the cuboid shape-matching loss is given by

$$L_\mathrm{shape}^\mathrm{cub} = \frac{1}{N}\sum_{i=1}^{N}\left\|\mathrm{ReLU}(\mathbf{p}_{\min} - \mathbf{P}_\mathrm{r}^{(i)}) + \mathrm{ReLU}(\mathbf{p}_{\max} - \mathbf{P}_\mathrm{r}^{(i)})\right\|_2^2, \tag{S12}$$

where N is the number of sampling points. This formulation ensures that no penalty is applied within the target spatial domain, while constraint violations are penalized smoothly once activated by the points that transcend the boundary. The resulting shape matching loss is differentiable, enabling stable and efficient training.

For the cylindrical domain illustrated in **Figure 4B**, whose axis is aligned with the $\mathbf{E}_1$-direction of the global frame defined in **Figure 4A** of the main text, the in-plane central point C is located at (c_2, c_3), the radius distance between the ring sampling points and the point C is determined by

$$\rho_i = \sqrt{(p_{\mathrm{r},2}^{(i)} - c_2)^2 + (p_{\mathrm{r},2}^{(i)} - c_3)^2}. \tag{S13}$$

With the application of the ReLU function, the final cylindrical domain shape-matching loss function is expressed as

$$L_\mathrm{shape}^\mathrm{cyl} = \frac{1}{N}\sum_{i=1}^{N}\left\{\begin{array}{l}\mathrm{ReLU}\left[\sqrt{(p_{\mathrm{r},2}^{(i)} - c_2)^2 + (p_{\mathrm{r},3}^{(i)} - c_3)^2} - R\right]^2 + \\ \mathrm{ReLU}\left[\mathrm{ReLU}(p_{1,\min} - P_{\mathrm{r},1}^{(i)}) + \mathrm{ReLU}(p_{1,\max} - P_{\mathrm{r},1}^{(i)})\right]^2\end{array}\right\}. \tag{S14}$$

The resulting loss vanishes when all sampled points lie within the target domain and increases quadratically with the magnitude of any boundary violation.

Shape-matching loss for conforming to a target curved surface

For the inverse design of a ring conforming to a target curved surface, shape matching is evaluated by the distance between the target surface and the ring configuration. To establish a general differentiable method for calculating distance, a point-to-surface distance loss based on the moving least squares (MLS) method is applied [5]. Suppose that the point set representing the ring configuration is given by $\mathbf{P} = \{\mathbf{p}_i\}_{i=1}^{N}$, $\mathbf{p}_i \in \mathbb{R}^3$, and that the point cloud of the target curved surface is denoted by $\mathbf{P}_{\text{target}} = \{\mathbf{t}_j\}_{j=1}^{M}$, $\mathbf{t}_j \in \mathbb{R}^3$. For each prediction point $\mathbf{p}_i$, the Euclidean distances to all target points $\mathbf{t}_j$ are computed, and the *k* nearest points are selected to form the *k*-nearest neighbor set $\mathbf{\Phi}_i = \{t_{i,1}, t_{i,2}, ..., t_{i,k}\} \subset \mathbf{P}_{\text{target}}$. For the neighborhood point set $\mathbf{\Phi}_i$, the geometric center $\boldsymbol{\mu}_i \in \mathbb{R}^3$ is calculated by

$$\boldsymbol{\mu}_i = \frac{1}{k}\sum_{j=1}^{k}\mathbf{t}_{i,j}. \tag{S15}$$

The local covariance matrix of the neighborhood is then established after decentration, as

$$\mathbf{C}_i = \frac{1}{k}\sum_{j=1}^{k}(\mathbf{t}_{i,j} - \boldsymbol{\mu}_i)(\mathbf{t}_{i,j} - \boldsymbol{\mu}_i)^{\mathrm{T}}. \tag{S16}$$

The core function of the covariance matrix is to quantify the distribution pattern of the local point cloud in 3D space, including its principal directions and degree of dispersion. To extract local geometric features, eigenvalue decomposition is applied to the symmetric covariance matrix $\mathbf{C}_i$. The resulting eigenvalues quantify the spatial variance of the local point cloud along their respective eigenvectors. Assuming that the points within the local neighborhood approximate a planar surface, the direction of minimum variance corresponds to the normal of the fitted local tangent plane. This relationship is mathematically expressed as

$$\mathbf{C}_i\mathbf{v}_i = \lambda_{\min}\mathbf{v}_i, \tag{S17}$$

where $\lambda_{\min}$ denotes the smallest eigenvalue, and its associated eigenvector $\mathbf{v}_i \in \mathbb{R}^3$ represents the estimated local surface normal.

With the local surface normal $\mathbf{v}_i$ determined, the signed point-to-plane distance d_i from the predicted ring configuration point p_i to its corresponding local tangent plane is computed. Specifically, the signed projection distance d_i is obtained by projecting the relative position vector onto the normal direction,

$$d_i = (\mathbf{p}_i - \boldsymbol{\mu}_i)\cdot\mathbf{v}_i. \tag{S18}$$

The geometric discrepancy is quantified using the squared point-to-plane distance, and the MLS-based shape-matching loss over the entire set of N predicted ring configuration points is defined as

$$L_{\text{shape}}^{\text{MLS}} = \frac{1}{N}\sum_{i=1}^{N} d_i^2. \tag{S19}$$

S4. Numerical evaluation of the KRT-PINN Framework

S4.1. Computational efficiency: KRT-PINN vs. FEM

To evaluate the computational efficiency, we compare the proposed KRT-PINN framework with FEM for the forward prediction of square rings with either constant or varying natural curvature, corresponding to the cases studied in **Figure 3**. The FEM models are constructed using shell elements. For consistency with the discretization adopted in the KRT-PINN framework, all FEM models use the same mesh settings, in which each rod segment is divided into 512 elements along the longitudinal direction and 5 elements along the width direction. As a result, each FEM model contains 10,340 elements in total.

The corresponding computational time statistics are summarized in **Table S2**. For the cases with available FEM results, the KRT-PINN framework using the Adam+SSBFGS optimization scheme consistently exhibits higher computational efficiency than FEM, particularly for rings with varying natural curvatures, where the computational time is reduced by 30%-60%. Note that the FEM computational time for the second stable state at $|\kappa_{\text{n2}}|L/2\pi$ = 0.5 is not reported in **Table S2**. This is because this stable state cannot be obtained by directly increasing the natural curvature from the planar initial configuration in FEM. Instead, additional prescribed perturbations, such as imposed rotations or displacements, are required to trigger the transition from the first stable state. To maintain a fair comparison under the same initialization strategy, this case was therefore excluded from the FEM timing comparison. Overall, for the forward prediction cases examined here, the KRT-PINN framework solved with an Adam+SSBFGS optimization scheme demonstrates a clear computational-efficiency advantage over FEM while preserving accurate prediction capability.

S4.2. Computational efficiency of the KRT-PINN framework for different rod systems

The computational efficiency of the KRT-PINN framework is further evaluated for different rod systems, including 2D segmented rings and 3D rod systems. For the 2D segmented rings, square, pentagon, and hexagonal rings are considered, with the total perimeter fixed at 400 mm. For the 3D rod systems, tetrahedral and cubic rod systems are considered, and the length of each rod segment was set to 100 mm. The KRT-PINN is then used to predict the stable

states of these 2D rings and 3D rod systems composed of rod segments with a dimensionless natural curvature of $\kappa_{n2}L/2\pi$ = 0.5. For each case, three independent runs are performed to evaluate the run-to-run variation and obtain the average computational time. For a segmented ring with n rod segments, the configuration of each rod segment is represented by independent rod-level neural networks. In the implementation, these independent rod networks are evaluated in a batched form, where the leading tensor dimension indexes the rod segment. This batching accelerates computation but does not introduce parameter sharing among rod segments. The model parameter scale still grows approximately linearly with rod number, while the batched computation avoids Python-loop overhead rod by rod and improves hardware utilization.

Table S3 summarizes the number of rod segments, number of joints, and average computational times for forward prediction across different rod systems using the KRT-PINN framework. For the tetrahedral and cubic rod systems, the reported computational times correspond to convergence to the first stable state, similar to the configuration shown in **Figure 6**. In general, as the number of rod segments increases, more trainable and nodal variables are introduced, leading to higher computational cost. However, the computational cost is not determined solely by the number of rod segments. For example, the tetrahedron rod system requires only approximately one quarter of the computational time needed for the hexagonal ring, although both systems contain six rod segments. This result indicates that the system topology and associated optimization complexity also influence the computational efficiency of the KRT-PINN framework.

S4.3. Comparison between KAN-based and MLP-based PINNs

Finally, the convergence behavior, training stability, prediction accuracy, and parameter efficiency of the current KAN-based PINN are compared with those of the conventional MLP-based PINN. For this comparison, an MLP-based PINN is constructed for each rod segment using a four-layer fully connected architecture, with 32 neurons in each hidden layer. The KAN-based PINN for each rod segment consists of a single cubic-spline KAN layer mapping the scalar arclength coordinate to 16 hidden features using 65 spline basis functions, followed by a fully connected output layer that predicts the four temporary quaternion components. Each of them is used to calculate the stable configuration of the square rings with constant or varying natural curvature in **Figure 3**. The training processes of both the PINN models are set as 500 Adam epochs followed by 1200 SSBFGS epochs to ensure the same training process. For each square ring, both the KAN-based and MLP-based PINNs are trained independently three times to study the convergence speed and the training stability. The corresponding training loss history comparisons of the ring under constant and varying natural curvature are summarized

in **Figure S5** and **Figure S6**, respectively. In each subplot, the solid red curve represents the mean loss history of the KAN-based PINN, while the dashed blue curve represents that of the MLP-based PINN. The shaded regions indicate the min–max envelope obtained from three independent training runs, thereby reflecting the run-to-run variability and convergence stability of each model. The central curve represents the mean loss history. A narrower shaded region generally indicates better training stability.

For the constant natural curvature cases shown in **Figure S5**, the KAN-based PINN generally exhibits more stable convergence than the MLP-based PINN, including $|\kappa_{n2}|L/2\pi = 0.5$ with two stable states and $|\kappa_{n2}|L/2\pi=1.0$. After the transition from Adam to SSBFGS optimization, marked by the vertical dotted line, the KAN-based PINN continues to reduce the loss more effectively, whereas the MLP-based PINN tends to approach a relatively flat plateau at an earlier stage. As a result, the KAN-based PINN achieves lower final total loss in all three cases. Its final strain energy loss is also lower in most cases, while in the first case, $|\kappa_{n2}|L/2\pi = 0.5$ (stable state ①), the final strain-energy losses of the two models are close, with the KAN-based PINN remaining slightly lower. The closure error of the KAN-based PINN also converges to a comparable or lower level in the final stage. Furthermore, the shaded regions of the KAN-based results are generally narrower after convergence, suggesting improved training stability and reduced sensitivity to initialization.

Figure S6 shows the same comparison for square rings with varying natural curvature, including the Euler spiral, cardioid, and sigmoidal curve cases. The KAN-based PINN again exhibits smoother convergence than the MLP-based PINN. After the transition from Adam to SSBFGS optimization, the KAN-based PINN continues to decrease the loss more effectively and achieves a lower final total loss in all three cases. The final strain energy loss is also lower in most cases. For the cardioid case, both models converge well, while the KAN-based PINN still attains a slightly lower value. The closure error histories also show that both models can reduce the closure loss effectively. In addition, the shaded regions of the MLP-based results are generally wider, showing larger variation across repeated runs, whereas the KAN-based results are more concentrated. These results demonstrate that the KAN-based PINN improves both convergence stability and final prediction accuracy for the current ring origami problems.

The comparisons of the predicted stable configurations are shown in **Figure S7**. The predictions of the KAN-based PINN are generally closer to the experimental results than the MLP-based PINN for both square rings with constant and varying natural curvatures. For the constant natural curvature cases in **Figure S7A**, both frameworks well predict the stable configuration of the case $|\kappa_{n2}|L/2\pi = 0.5$ (stable state ①), whereas for the other two cases the

KAN-based predictions are noticeably closer to the experiments and the MLP-based PINN shows larger deviations (marked in the dashed box). For the varying natiural curvature cases in **Figure S7B**, both frameworks predict the cardioid case reasonably well, while for the Euler spiral and sigmoidal-curve cases the MLP-based PINN deviates more significantly from the experimental results, whereas the KAN-based PINN continues to produce good agreement with the experiments. These comparisons further represent that the KAN-based PINN achieves better overall prediction accuracy than the MLP-based PINN.

To quantify parameter efficiency, we count all trainable degrees of freedom in the rod networks under identical geometric and loss settings, as summarized in **Table S4**. The KAN-based network contains 1,140 trainable parameters per rod, compared with 3,364 for the MLP-based network. For the four-rod square ring, the total number of trainable rod-network parameters is 4,560 for the KAN-PINN and 13,456 for the MLP-PINN, meaning that the MLP-based framework has about 2.95 times as many rod-network parameters as the KAN-based framework. After including the shared trainable nodal variables, the total trainable parameter count becomes 4,588 for the KAN-PINN, which remains substantially lower than 13,484 for the MLP-PINN.

Overall, for the ring origami problems studied in this work, the KAN-based PINN exhibits better convergence behavior, higher prediction accuracy, improved training stability, and greater parameter efficiency than the MLP-based PINN.

Supplementary figures

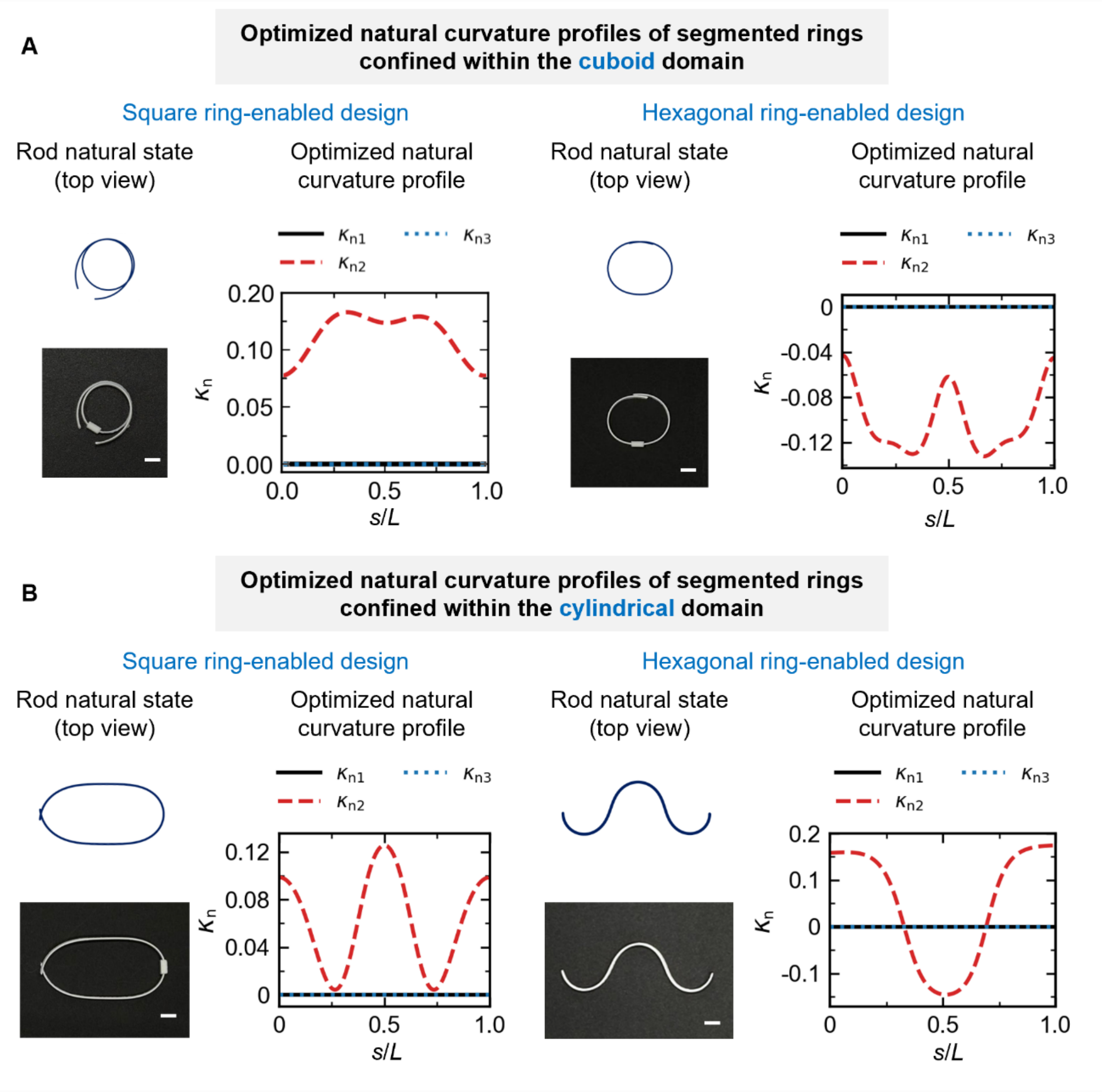

Figure S1. Optimized natural curvature profiles of rod segments in segmented rings confined within prescribed spatial domains. **A**) Cuboid domain: Rod natural states and corresponding natural curvature profiles for the square ring-enabled design (left) and hexagonal ring-enabled design (right). **B**) Cylindrical domain: Rod natural states and corresponding natural curvature profiles for the square ring-enabled design (left) and hexagonal ring-enabled design (right).

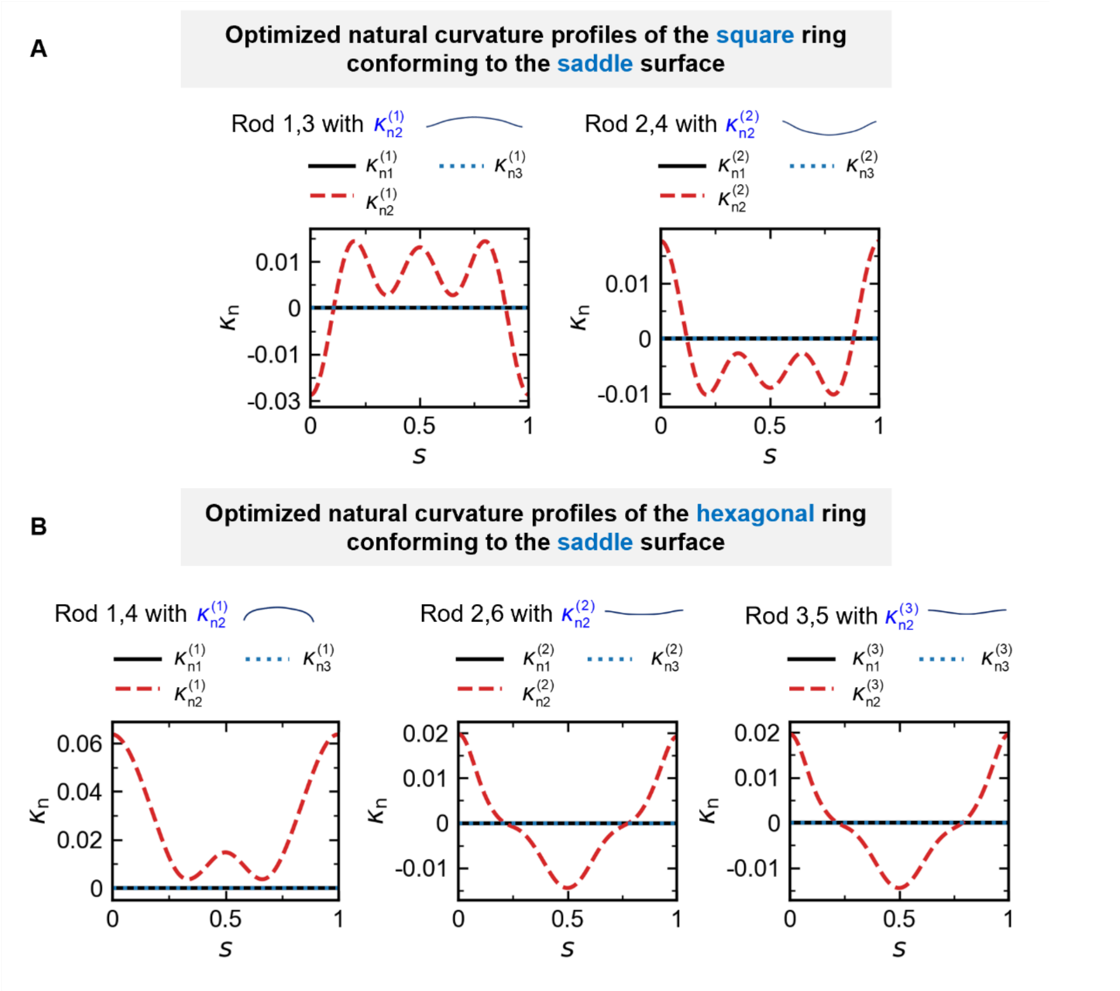

Figure S2. Optimized natural curvature profiles of rod segments in segmented rings conforming to the saddle surface. **A)** Optimized natural curvature profiles of the two pairs of rod segments in the square ring shown in Figure 5A of the main text. **B)** Optimized natural curvature profiles of the three pairs of rod segments in the hexagonal ring shown in Figure 5A of the main text.

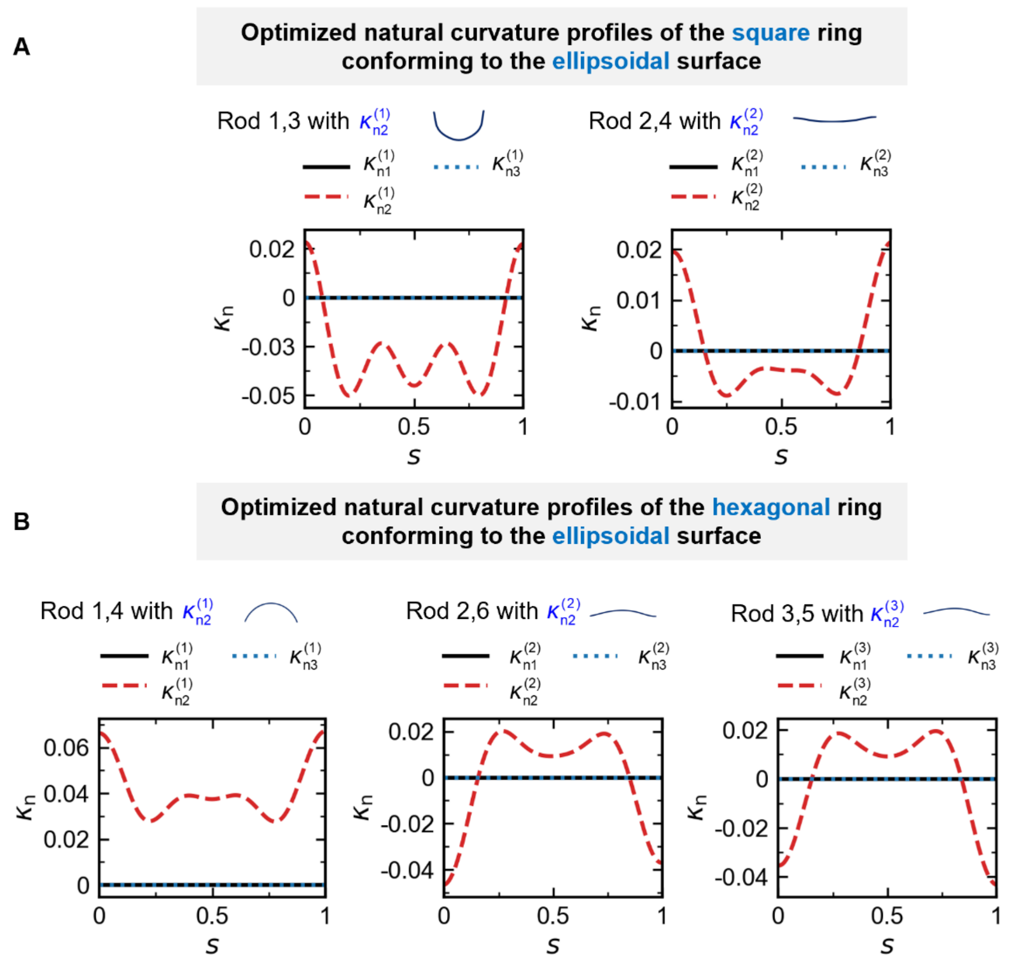

Figure S3. Optimized natural curvature profiles of rod segments in segmented rings conforming to the ellipsoidal surface. **A)** Optimized natural curvature profiles of the two pairs of rod segments in the square ring shown in Figure 5A of the main text. **B)** Optimized natural curvature profiles of the three pairs of rod segments in the hexagonal ring shown in Figure 5A of the main text.

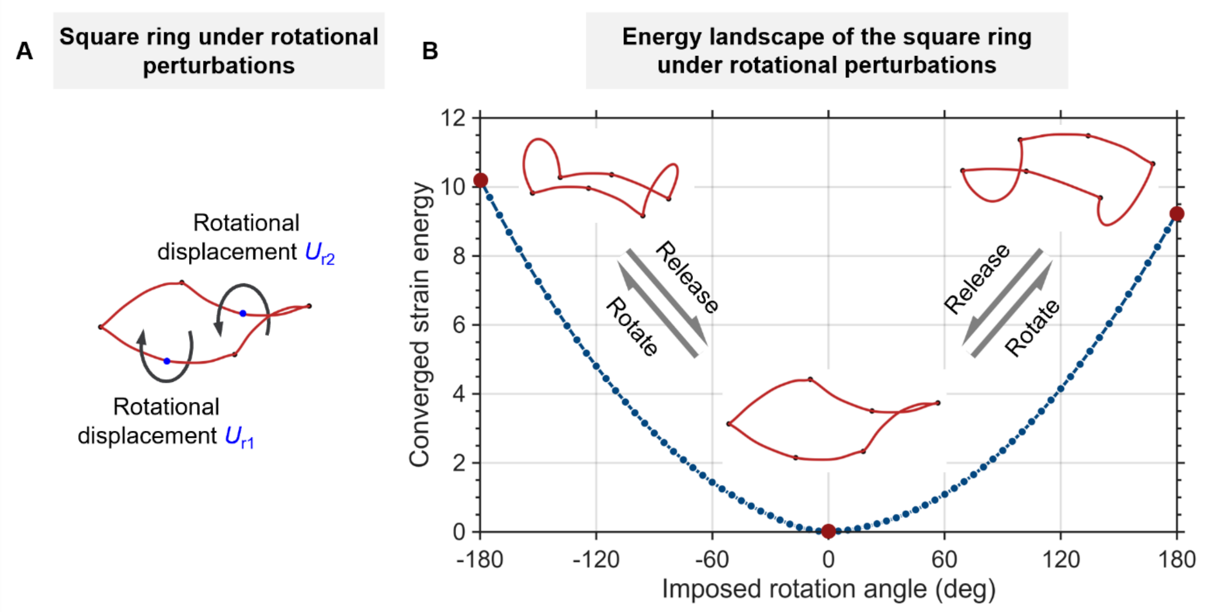

Figure S4. Stability assessment of the designed square ring conforming to a saddle surface under rotational perturbations. **A**) Schematic of the square ring under an imposed perturbation, where a pair of opposite rotational displacements with equal magnitude is applied at the midpoints of two opposite rod segments. **B**) Energy landscape of the square ring under the rotational perturbations. The unperturbed stable state corresponds to the minimum strain energy. Once the perturbation is removed, the ring spontaneously returns to its stable minimum-energy state.

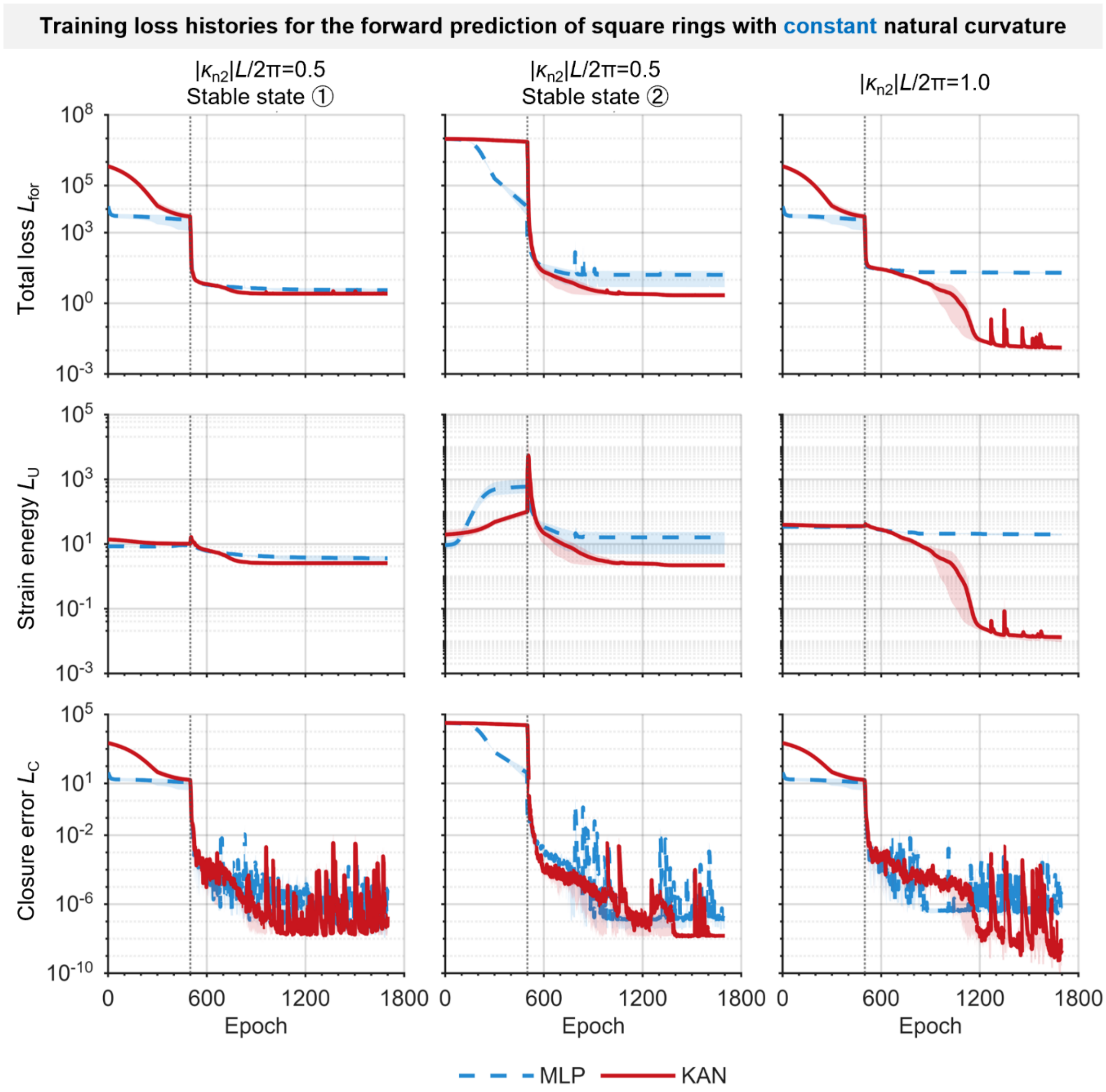

Figure S5. Comparison of training loss histories between the KAN-based and MLP-based PINNs for the forward prediction of square rings with constant natural curvature studied in **Figure 3A**. Three loss components are presented: total loss L_{for} (top), strain energy L_{U} (middle), and closure error L_{C} (bottom). The vertical dashed line marks the transition from Adam to SSBFGS optimization. The solid red curves denote the KAN-based PINN, and the dashed blue curves denote the MLP-based PINN. The shaded regions indicate the run-to-run variation, defined by the minimum and maximum loss values among three independent training runs at each epoch, while the central curve represents the mean loss history.

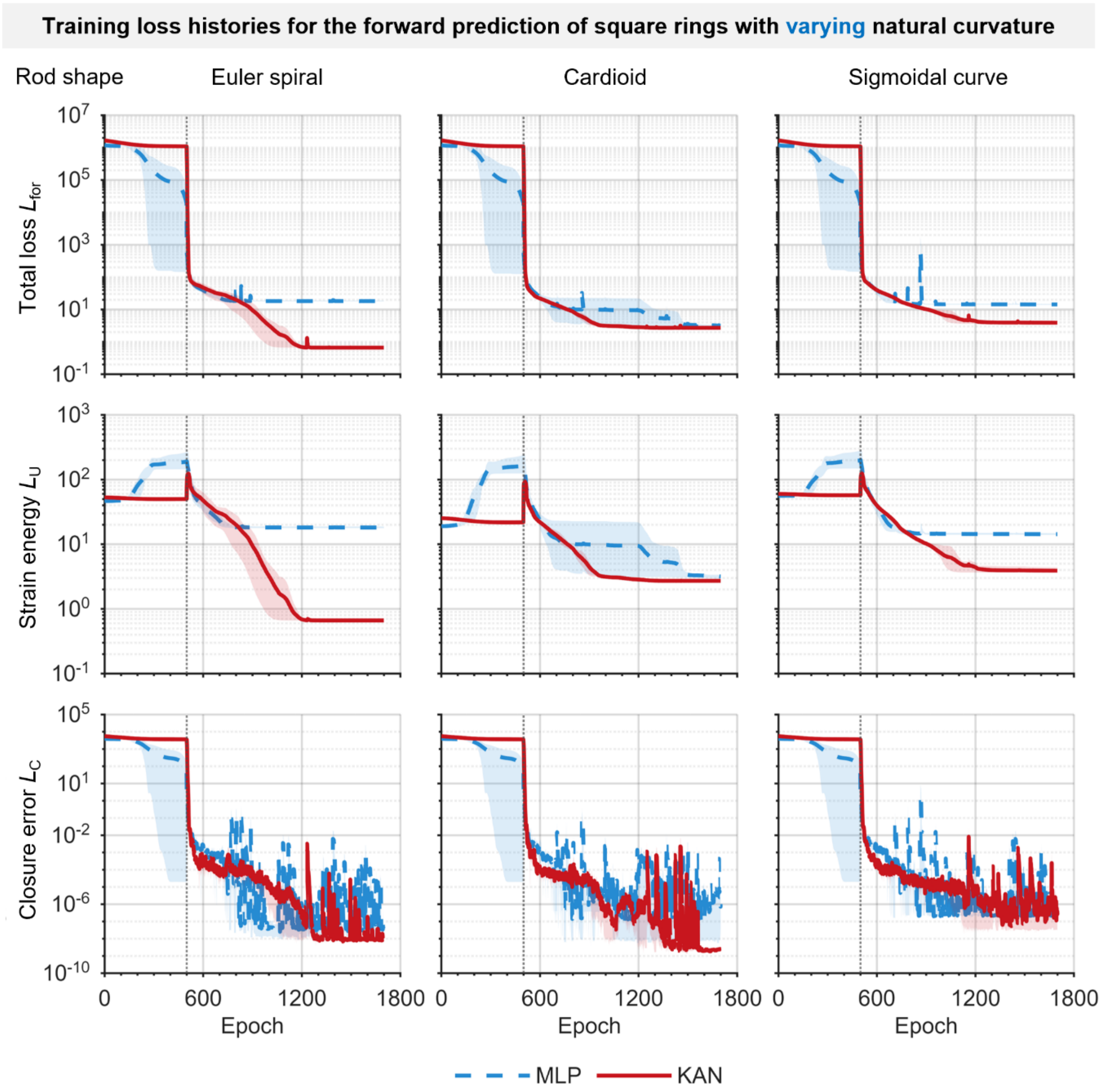

Figure S6. Comparison of training loss histories between the MLP-based and KAN-based PINNs for the forward prediction of square rings with varying natural curvature studied in **Figure 3**B. Three loss components are presented: the total loss L_{for} (top), strain energy L_U (middle), and closure error L_C (bottom). The vertical dashed line marks the transition from Adam to SSBFGS optimization. The solid red curves denote the KAN-based PINN, and the dashed blue curves denote the MLP-based PINN. The shaded regions indicate the run-to-run variation, defined by the minimum and maximum loss values among three independent training runs at each epoch, while the central curve represents the mean loss history.

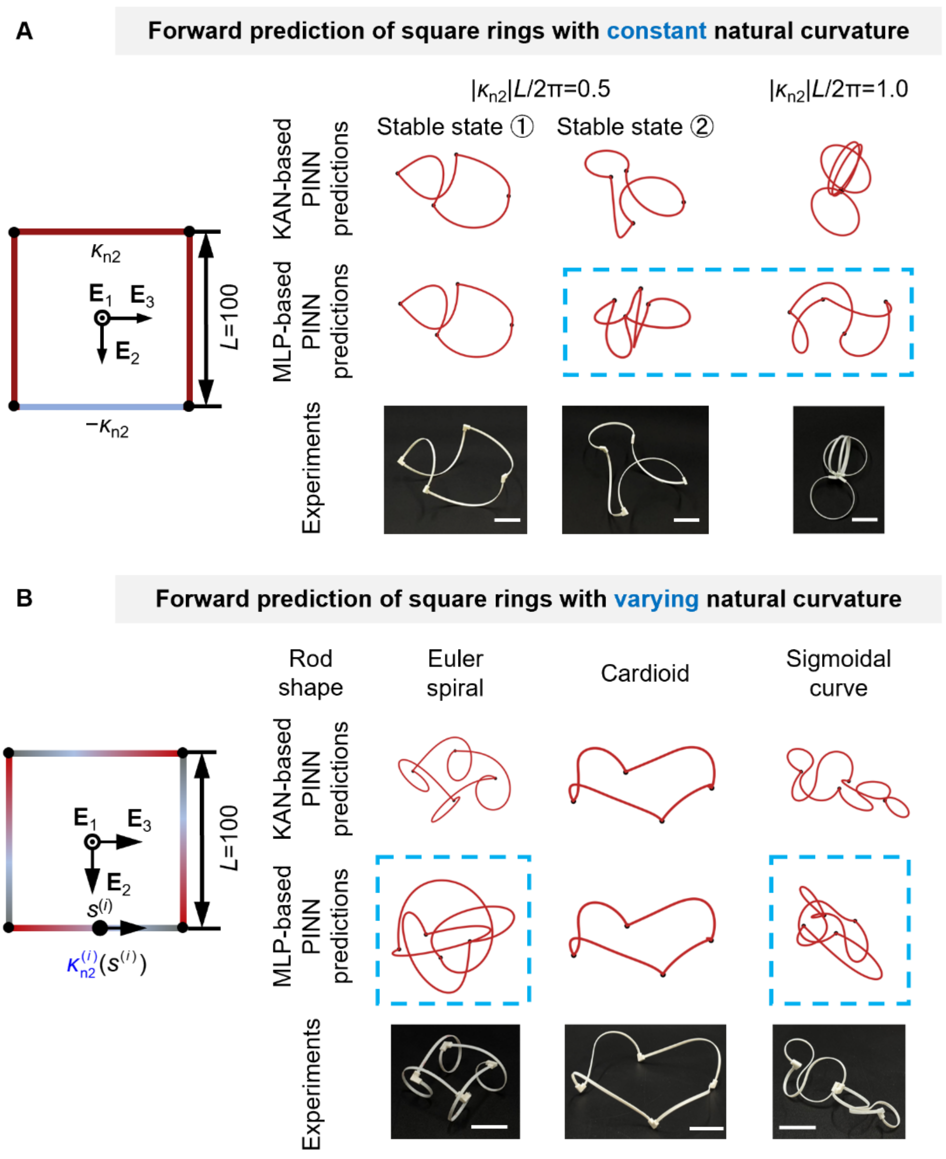

Figure S7. Comparison between the MLP-based and KAN-based PINNs for forward prediction of stable states of square rings studied in **Figure 3**. **A**) Square rings with constant natural curvature: (Left) Schematic of a square ring composed of three rod segments with positive constant natural curvature κ_{n2} and one rod segment with negative constant natural curvature $-\kappa_{n2}$, where rod segments with positive and negative natural curvature are marked in red and blue, respectively. (Right) Stable states of the rings with constant dimensionless natural curvature $|\kappa_{n2}|L/2\pi = 0.5$ and 1.0, predicted by the KAN-based PINN, MLP-based PINN, and validated experimentally. **B**) Square rings with varying natural curvature: (Left) Schematic of a square ring composed of rod segments sharing the same varying natural curvature. (Right) Stable states of square rings composed of rod segments with different prescribed natural shapes, including an Euler spiral, a cardioid, and a sigmoidal curve, predicted by the KAN-based PINN, MLP-based PINN, and validated experimentally. Scale bars: 20 mm.

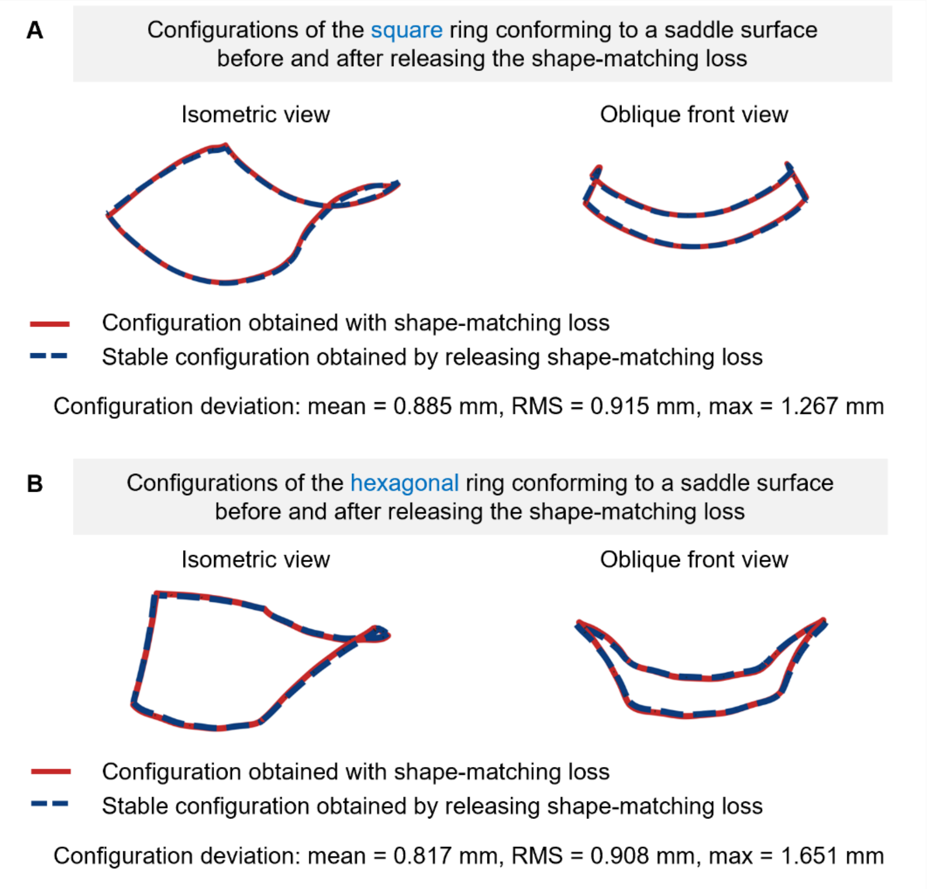

Figure S8. Comparison of the inversely designed configurations before and after releasing the shape-matching loss for **A)** square and **B)** hexagonal rings conforming to a saddle surface. In each case, the red solid curve denotes the configuration obtained after the first-stage optimization with the shape-matching loss, and the blue dashed curve denotes the final stable configuration obtained after the second-stage optimization without the shape-matching loss. The configuration deviation is quantified by the mean, Root-Mean-Square (RMS), and maximum nodal deviations.

Supplementary tables

Table S1. Coefficients of the prescribed nonuniform natural curvature profile defined by Equation (6) for the forward prediction of the square ring in **Figure 3B**.

Coefficients	Linear terms		Fourier series terms					
	a	b	c_1	c_2	c_3	d_1	d_2	d_3
Euler spiral	0	1.26e-1	0	0	0	0	0	0
Cardioid	-1.23e-1	1.93e-1	2.01e-2	2.09e-3	7.80e-3	2.86e-2	1.80e-2	8.65e-3
Sigmoidal curve	0	0	0	0	1.00e-2	1.07e-1	0	3.56e-2

Table S2. Comparison of computational time (s) between the KRT-PINN framework and FEM for square rings with different natural curvature profiles.

Natural curvature profile	KRT-PINN (Adam + SSBFGS)	FEM
$\lvert\kappa_{n2}\rvert L/2\pi = 0.5$ (Stable state ①)	62.3	97.3
$\lvert\kappa_{n2}\rvert L/2\pi = 0.5$ (Stable state ②)	101.2	-
$\lvert\kappa_{n2}\rvert L/2\pi = 1.0$	120.8	127.7
Euler spiral	98.5	295.0
Cardioid	101.1	148.5
Sigmoidal curve	113.7	235.5

Table S3. Comparison of forward prediction computational times of the KRT-PINN framework for different 2D rings and 3D rod systems.

	Square ring	Pentagonal ring	Hexagonal ring	Tetrahedral rod system	Cubic rod system
Rod segment number	4	5	6	6	12
Joint number	4	5	6	4	8
Average computational time (s)	76.6	150.3	232.0	53.3	117.3

Table S4. Comparison of trainable parameters between the KAN-based and MLP-based PINN under identical geometric and loss settings.

	Parameters per rod	Four-rod network parameters	Trainable nodal variables	Total trainable parameters
KAN-based PINN	1,140	4,560	28	4,588
MLP-based PINN	3,364	13,456	28	13,484

References

[1] B. Audoly, and Y. Pomeau, *Elasticity and geometry: from hair curls to the non-linear response of shells*: Oxford University Press, 2010.

[2] T. J. Healey, and P. Mehta, “Straightforward computation of spatial equilibria of geometrically exact cosserat rods,” *International Journal of Bifurcation and Chaos,* vol. 15, no. 03, pp. 949-965, 2005.

[3] T. Yu, and J. Hanna, “Bifurcations of buckled, clamped anisotropic rods and thin bands under lateral end translations,” *Journal of the Mechanics and Physics of Solids,* vol. 122, pp. 657-685, 2019.

[4] S. Timoshenko, and J. N. Goodier, *Theory of Elasticity: By S. Timoshenko and JN Goodier*: McGraw-Hill, 1951.

[5] M. Alexa, J. Behr, D. Cohen-Or, S. Fleishman, D. Levin, and C. T. Silva, "Point set surfaces." *Proceedings Visualization*, pp. 21-29, 537, 2001.
